# Novel RPTPγ and RPTPζ splice variants from mixed neuron–astrocyte hippocampal cultures as well as from the hippocampi of newborn and adult mice

**DOI:** 10.3389/fphys.2024.1406448

**Published:** 2024-06-17

**Authors:** Sara Taki, Walter F. Boron, Fraser J. Moss

**Affiliations:** ^1^ Department of Physiology and Biophysics, Case Western Reserve University School of Medicine, Cleveland, OH, United States; ^2^ Department of Medicine, Case Western Reserve University School of Medicine, Cleveland, OH, United States; ^3^ Department of Biochemistry, Case Western Reserve University School of Medicine, Cleveland, OH, United States

**Keywords:** astrocyte expression, central nervous system, CO_2_, HCO_3_-sensing, gene variants, mouse hippocampus, receptor protein tyrosine phosphatase (RPTP) γ and ζ, neuronal expression

## Abstract

Receptor protein tyrosine phosphatases γ and ζ (RPTPγ and RPTPζ) are transmembrane signaling proteins with extracellular carbonic anhydrase–like domains that play vital roles in the development and functioning of the central nervous system (CNS) and are implicated in tumor suppression, neurodegeneration, and sensing of extracellular [CO_2_] and [HCO_3_^−^]. RPTPγ expresses throughout the body, whereas RPTPζ preferentially expresses in the CNS. Here, we investigate differential RPTPγ-RPTPζ expression in three sources derived from a wild-type laboratory strain of C57BL/6 mice: (a) mixed neuron–astrocyte hippocampal (HC) cultures 14 days post isolation from P0–P2 pups; (b) P0–P2 pup hippocampi; and (c) 9- to 12-week-old adult hippocampi. Regarding RPTPγ, we detect the *Ptprg* variant-1 (V1) transcript, representing canonical exons 1–30. Moreover, we newly validate the hypothetical assembly [XM_006517956] (propose name, *Ptprg*-V3), which lacks exon 14. Both transcripts are in all three HC sources. Regarding RPTPζ, we confirm the expression of *Ptprz1*-V1, detecting it in pups and adults but not in cultures, and *Ptprz1*-V3 through *Ptprz1*-V7 in all three preparations. We newly validate hypothetical assemblies *Ptprz1*-X1 (in cultures and pups), *Ptprz1*-X2 (in all three), and *Ptprz1*-X5 (in pups and adults) and propose to re-designate them as *Ptprz1*-V0, *Ptprz1*-V2, and *Ptprz1*-V8, respectively. The diversity of RPTPγ and RPTPζ splice variants likely corresponds to distinct signaling functions, in different cellular compartments, during development vs later life. In contrast to previous studies that report divergent RPTPγ and RPTPζ protein expressions in neurons and sometimes in the glia, we observe that RPTPγ and RPTPζ co-express in the somata and processes of almost all HC neurons but not in astrocytes, in all three HC preparations.

## Introduction

The human protein tyrosine phosphatase proteome (PTPome) comprises about 125 proteins (Alonso et al., 2004; Alonso and Pulido, 2016), encoded by 39 genes. Of these 39 genes, 17 encode cytosolic or non-transmembrane (PTPN) proteins and 22 encode receptor (PTPR) or transmembrane proteins (Alonso et al., 2004; Lee et al., 2015; Alonso and Pulido, 2016). The protein tyrosine phosphatases (PTPs) are implicated in the regulation of gene transcription, mRNA processing, control of mitosis, cell differentiation, cell growth, and receptor-mediated endocytosis, as well as in sensing the extracellular CO_2_/HCO_3_^−^ concentrations (Tonks, 2013; Zhou et al., 2016; Young et al., 2021; Zhang et al., 2023). Abnormalities in PTPs play an important role in the pathogenesis of numerous diseases, from cancer to immune deficiency (He et al., 2014). The loss of function of certain PTPs, such as PTPN13 (Mcheik et al., 2020), PTEN (González-García et al., 2022), PTPRD (Szaumkessel et al., 2017), and the receptor protein tyrosine phosphatases γ (RPTPγ or PTPRG) and ζ (RPTPζ or PTPRZ), can contribute to cancer progression (Boni and Sorio, 2021; Sloth et al., 2022).

RPTPγ and RPTPζ comprise the R5 (or class V) PTP subfamily (Alonso et al., 2004), which is distinguished by (a) an amino-terminal (Nt) extracellular carbonic anhydrase–like domain (CALD) that sequentially links to (b) a single extracellular fibronectin III (FNIII) domain, (c) a transmembrane (TM) domain, (d) the D1 PTPase domain, (e) the D2 blocking domain, and (f) a PDZ-binding domain at the cytosolic carboxy terminus (Ct) (Krueger and Saito, 1992; Barnea et al., 1993). Both RPTPγ and RPTPζ have multiple splice variants, which include secreted proteins (i.e., they lack the TM domain).

Within RPTPγ and RPTPζ, the substitution of residues conserved in the α-carbonic anhydrases (α-CAs), specifically the solvent network, proton shuttle, and two of the three conserved histidine residues that coordinate Zn^2+^, render the CALDs catalytically inactive. However, the CALD retains most of the other amino acids that are highly conserved in α-CAs, suggesting to us that the CALDs can still bind CO_2_ or HCO_3_^−^, even if they cannot catalyze CO_2_/HCO_3_^−^ interconversion (Zhou et al., 2016).

In addition, CO_2_/HCO_3_^−^, RPTPγ and RPTPζ CALDs also bind to contactin (CNTN) cell-adhesion molecules. The RPTPγ CALD binds to the second and third Ig repeats in CNTN3, 4, 5, or 6 via an extended β-hairpin loop (residues 288–301) not present in the α-CAs, with the remaining contacts provided by RPTPγ residues 225–229. In the RPTPζ CALD, a homologous β-hairpin loop (residues 267–280), with additional contacts from R208 and K208, binds to Ig domains 2 and 3, but only of CNTN1 (Peles et al., 1995; Bouyain and Watkins, 2010; Lamprianou et al., 2011). The differences between the key amino acid residues in the RPTPζ CALD β-hairpin loop vs RPTPγ largely explain the specificity of the RPTPζ-CNTN1 interaction (Bouyain and Watkins, 2010; Lamprianou et al., 2011). These CALD-contactin interactions promote cell adhesion during the development and maintenance of the central nervous system (CNS), and their disruption is implicated in neurological disorders, such as autism and schizophrenia (Fernandez et al., 2004; Roohi et al., 2009; Lamprianou et al., 2011; Oguro-Ando et al., 2017).

Another RPTPζ ligand is pleiotrophin (PTN), a heparin-binding growth factor involved in cell proliferation, differentiation, and migration (Mohebiany et al., 2013). Yet a third are the chondroitin sulfates that anchor to numerous serine–glycine dipeptide motifs located between the FNIII and TM domains of RPTPζ (Maurel et al., 1994). Depending on their structure, the chondroitin sulfates tend to increase the binding affinity of PTN to the RPTPζ extracellular domain (Maeda et al., 1996). PTN binding induces RPTPζ dimerization, which includes trans-protomer D1–D2 interactions that block substrate access to the active sites of the two intracellular phosphatase domains. The net result is increased tyrosine phosphorylation (Fukada et al., 2006) due to the unbalanced action of tyrosine kinases of various downstream signaling molecules of RPTPζ, which include, but are not limited to, β-catenin (Meng et al., 2000; Perez-Pinera et al., 2006), G protein–coupled receptor kinase interactor 1 (Kawachi et al., 2001; Fukada et al., 2005), and anaplastic lymphoma kinase (Perez-Pinera et al., 2007). We are not aware of any reports of PTN binding to or inducing dimerization of RPTPγ.

RPTPγ, for which the full-length human and mouse cDNAs were first cloned by the Schlessinger laboratory (Barnea et al., 1993), is almost ubiquitously expressed in mammalian tissues. RPTPγ expression levels are particularly high in the CNS, endocrine, immune, and various epithelial or endothelial tissues, particularly in the lungs, liver, and kidneys (Tsukamoto et al., 1992; Barnea et al., 1993; Lamprianou et al., 2006; Vezzalini et al., 2007; Lorenzetto et al., 2014; Uhlén et al., 2015; Hansen et al., 2020). In the CNS, RPTPγ is present in the embryonic brain, where it regulates neurite outgrowth and cell adhesion (Sahin et al., 1995; Shintani et al., 2001). In the adult brain, RPTPγ predominantly localizes to the neurons of the neocortex, striatum, cerebellum, many nuclei of the brainstem, and the hippocampus stratum pyramidale (SP) (Lamprianou et al., 2006; Lorenzetto et al., 2014). In the cerebellum, Bergmann radial glia, as identified by co-staining with either glial fibrillary acidic protein (GFAP) or S100 EF-hand Ca^2+^-binding protein (Lorenzetto et al., 2014), express RPTPγ. Resting astrocytes and microglia *in situ* in the adult mouse brain are almost always negative for RPTPγ expression (Lamprianou et al., 2006; Lorenzetto et al., 2014). To summarize from previous reports, RPTPγ expresses widely throughout the body; in the CNS, RPTPγ is present mainly in certain neurons and in some astrocytes.

Schlessinger and collaborators generated RPTPγ knockout (*Ptprg* ^−/−^) mice (Lamprianou et al., 2006). These mice develop normally, exhibit only mild behavioral abnormalities (Lamprianou et al., 2006), but have a marked decrease in their ability to defend against chronic whole-body metabolic acidosis (Zhou et al., 2016). *Ptprg* ^−/−^ mice also exhibit an attenuated vasodilator response when extracellular HCO_3_^−^ concentration ([HCO_3_^−^]_o_) decreases (Boedtkjer et al., 2016) and are prone to elevated blood pressure induced by hyperventilation (Hansen et al., 2020). Moreover, loss-of-function mutations and single-nucleotide polymorphisms in human RPTPγ are also associated with heart ischemic vascular disease (Hansen et al., 2020) and increased risk of human cerebral infarction (Carty et al., 2015).

Saito and colleagues were the first to clone the cDNA encoding full-length RPTPζ using human tissue (Krueger and Saito, 1992). Shortly thereafter, Schlessinger and collaborators cloned and characterized RPTPζ from both humans and mice (Levy et al., 1993). RPTPζ expresses in some peripheral tissues, such as stomach and bone (Schinke et al., 2008; Fujikawa et al., 2017; Fujikawa et al., 2019), as well as Schwann cells in the peripheral nervous system (Snyder et al., 1996). However, RPTPζ preferentially expresses in the CNS (Levy et al., 1993), which includes the spinal cord (Milev et al., 1994). High RPTPζ expression levels in the embryonic ventricular zone (VZ) and sub-ventricular zone (SVZ) are consistent with an important role for RPTPζ in CNS development (Levy et al., 1993; Canoll et al., 1996). During development, the radial processes of several classes of glia exhibit RPTPζ expression (Canoll et al., 1993; Canoll et al., 1996; Lorenzetto et al., 2014). RPTPζ splice variants with TM domains tend to be expressed in the glial progenitors located in the SVZ, whereas the secreted variant, phosphacan, is expressed at high levels in more mature astrocytes located beyond the SVZ (Canoll et al., 1996). Schwann cells and astrocytes that surround the olfactory bulb axons also express RPTPζ (Canoll et al., 1993), as do primary astrocytes in culture in rats (Sakurai et al., 1996), as well as glioblastoma (Krueger and Saito, 1992) and glioma cell lines (Sakurai et al., 1996). Furthermore, Shintani et al. (1998) reported astrocytic RPTPζ expression in both cortical cultures from e16 embryonic mice, early postnatal animals (≤P10), and in adult cortical and hippocampal (HC) astrocytes. To summarize from previous reports, RPTPζ expresses mainly in the CNS, particularly in cells of the VZ/SVZ and radial glial cells of embryos and in certain neurons in adults.

The considerable disparity in the reported levels of astrocytic RPTPζ expression among different studies is likely contingent upon several factors, which include (a) whether the astrocytes are cultured or *in situ*, (b) the specific brain region from which they originate, (c) whether they are quiescent or reactive, (d) their age, and (e) whether it is mRNA transcript or protein expression that is reported.

The Noda and Schlessinger laboratories independently generated RPTPζ knockout (*Ptprz1* ^−/−^) mice (Shintani et al., 1998; Harroch et al., 2000). Adult *Ptprz1* ^−/−^ mice of either strain show no obvious anatomical abnormalities (Shintani et al., 1998; Harroch et al., 2000; Tamura et al., 2006). However, they exhibit increased oligodendrocyte differentiation, impaired remyelination (Harroch et al., 2002; Kuboyama et al., 2012; Kuboyama et al., 2015; Kuboyama et al., 2016), and age-dependent impaired spatial learning (Niisato et al., 2005; Tamura et al., 2006).

In the present study, we examine (a) mixed neuron–astrocyte HC cultures from P0–P2 mice, and HC tissue from (b) P0–P2 neonates and (c) adults. We detect the mRNAs encoding and clone the corresponding cDNAs of both novel and previously reported mouse RPTPγ and RPTPζ variants. We also determine by immunocytochemistry (ICC) and immunohistochemistry (IHC) in each cell/tissue preparation that RPTPγ and RPTPζ co-express in the same mouse HC neurons. However, we detect neither RPTPγ nor RPTPζ in HC astrocytes. This observation corroborates much of the previous literature regarding the almost exclusive expression and localization of RPTPγ in neurons. However, the present results are in contrast to some previous studies reporting either that RPTPγ and RPTPζ express in different types of neurons (Lorenzetto et al., 2014) or that RPTPζ is primarily expressed in glia (Canoll et al., 1993; Canoll et al., 1996; Shintani et al., 1998; Harroch et al., 2000).

## Methods

### Ethical approval and animal procedures

Protocols for housing and handling mice were approved by the Institutional Animal Care and Use Committee at Case Western Reserve University.

### Mouse lines and breeding

The *Ptprg* ^−/−^ and *Ptprz1* ^−/−^ mice were generous gifts of Prof. Joseph Schlessinger (Harroch et al., 2000; Lamprianou et al., 2006). We backcrossed for more than seven generations with our standard laboratory wild-type (WT) strain, C57/BL6_Case_, which we derived from mice originally provided by Prof. Alan Verkman as heterozygotes for the aquaporin 1 knockout (*Aqp1* ^−/−^) mouse (Ma et al., 1998).

### Cell culture

We obtained primary co-cultures of HC neurons and astrocytes from WT and *Ptprz1* ^−/−^ mice as described previously (Bouyer et al., 2004; Salameh et al., 2014; Salameh et al., 2017). In summary, after decapitating non-anesthetized P0–P2 pups (both sexes), we identified and isolated the hippocampus and digested the tissue in a HEPES-buffered saline containing (in mM): 143.7 NaCl, 3 KCl, 10 HEPES, 1.1 EDTA, 5.5 L-cysteine, and 1% papain (cat# LS003162, Worthington Biochemical Corp., Lakewood, NJ), at pH 7.40. After 10 min of papain digestion at 37°C, to disperse cells, we triturated (using a series of flamed Pasteur pipettes of decreasing tip diameters, from 5 to 3 to 1 mm) the tissue in media containing 22 mM HCO_3_^−^, dissolved in 9.5% minimum essential medium (MEM; cat# 61100-103, Gibco, BRL, Life Technologies Inc., Gaithersburg, MD), 1.5% trypsin inhibitor (cat# T9253, Sigma-Aldrich, St. Louis, MO), and 1.5% bovine serum albumin (BSA, cat# A7906, Sigma-Aldrich) equilibrated with 5% CO_2_ in a 37°C incubator, at a pH of 7.40. After trituration, we diluted the cell suspension to a concentration of ∼1 × 10^5^ cells∙ml^−1^, plated this suspension onto a coverslip previously coated with 0.1% poly-L-ornithine, and placed it in a 35-mm Petri dish containing 1 mL culture media that contained 70% preconditioned complete media (see below), 30% neurobasal media (cat# 21103, Gibco), supplemented with 0.02% B27 (cat# 17504–044, GIBCO), 10 ng ml^−1^ FGF-5 (cat# F4537, Sigma-Aldrich), and 1 ng mL^−1^ basic fibroblast growth factor (cat# F0291, Sigma-Aldrich). We placed these Petri dishes in an incubator at 37°C, with 5% CO_2_/balanced air. After 24 h (and then every 2 days after that), we exchanged half of the media with neurobasal media supplemented with 10% B27 and 1% pen/strep. We used the cells for physiological studies between day 14 and ∼23 in culture.

The preconditioned complete media was made by incubating 200 μL of freshly dissociated cells (∼1 × 10^5^ cells∙ml^−1^) in 10 mL MEM solution supplemented with 10% fetal bovine serum (cat# 26140-079, Gibco) and 0.02% penicillin–streptomycin (cat# 15140–122, Gibco) for 1 week, followed by filtration.

### Total RNA isolation for RT-PCR

#### From cell culture

We aspirated the culture media from day 14, mixed neuron–astrocyte HC cultures, and added 0.3 mL TRIzol reagent (cat# 1559602, Thermo Fisher Scientific) per 35-mm dish. We homogenized the sample by repeated pipetting using a P-200 pipette tip and then followed the manufacturer’s instructions for phenol–chloroform extraction and ethanol precipitation to purify total RNA (TRNA). We dissolved the purified TRNA in RNAse-free H_2_O, assayed concentration and quality using a NanoDrop 2000 UV spectrophotometer (Thermo Fisher Scientific) to assess absorbance at 260 and 280 nm, and stored 5-μg/μL aliquots in single-use tubes at −80°C until ready to perform RT-PCRs.

#### From tissue

The initial steps for isolating pup HC tissue for TRNA isolation were as described above for the preparation of mixed neuron–astrocyte HC cultures. However, instead of digesting the isolated pup hippocampi, we immediately snap-froze and stored the tissue at −80°C until we collected enough samples to isolate TRNA with TRIzol reagent.

To isolate adult mouse hippocampi, we sacrificed 9- to 12-week-old animals under isoflurane-induced anesthesia by cervical dislocation. We immediately dissected the hippocampi from both hemispheres, snap-froze the samples, and then stored them at −80°C until we isolated TRNA with TRIzol reagent.

To isolate TRNA, we added 1 mL of TRIzol reagent to 10 frozen pup hippocampi or 5–6 frozen adult hippocampi and then homogenized for 5 min at room temperature. We then purified and stored the TRNA as described above for the cell culture samples.

### RT-PCR cloning

We followed the manufacturer’s instructions from the SuperScript IV First-Strand Synthesis System (cat# 18091050, Thermo Fisher Scientific) to perform reverse transcription from the TRNA isolated from culture, pup, or adult mouse hippocampi primed with 2.5 μM Oligo (dT)_20_ in a 20-µL total reaction volume. The RNA template was digested with RNase H, the cDNA product was isolated from the reaction using the QIAquick PCR Purification Kit (cat# 28104, Qiagen), and the cDNA concentration assayed by NanoDrop UV spectrophotometry (Thermo Fisher Scientific).

We assembled PCRs using 5 μg of cDNA template with PrimeSTAR HS DNA Polymerase (cat# R010A, Takara Bio), dNTPs, and the gene-specific primers according to the manufacturer’s instructions. We performed 30 cycles of PCR amplification, loaded the PCRs on 1% agarose gels, isolated amplified bands from the agarose with a sterile scalpel, and purified the cDNA fragments from the agarose using the QIAquick Gel Extraction Kit (cat# 28704, Qiagen). The concentration of each isolated band was determined using NanoDrop UV spectrophotometry. We ligated the PCR bands into the pCR-Blunt plasmid using the Zero Blunt PCR Cloning Kit (cat# K270040, Thermo Fisher Scientific) and transformed the plasmid into TOP-10 chemically competent *E. coli* provided in the kit. Individual colonies were isolated from Kanamycin-selective agar plates (50 μg/mL Kanamycin) and grown in LB media (+50 μg/mL Kanamycin) overnight at 37°C. We isolated and purified plasmid using the QIAprep Spin Miniprep Kit (cat# 27104, Qiagen), and the inserted PCR fragment was sequenced with M13 Forward, M13 Reverse, and other gene-specific primers.

#### PCR primers

For each primer, we provide the nomenclature, primer sequence, and location in Table 1.

**TABLE 1:** Nomenclature, sequence, and description of all PCR primers used in the present study.

Primer name	Primer sequence	Notes
γEx1_Fwd	5′-ATC​GTG​TCT​GAG​CGG​AAA​GC-3′	Corresponds to 5′-UTR sequence, 266 nt before the start codon in Exon 1
γEx14a_Fwd	5′-GAC​GTG​GAT​GCT​GGA​AAG​CTG-3′	Corresponding to nt 4–24 of exon 14a
γEx30_Rev	5′-GAC​CTT​CAC​TGC​ACG​GAA​CT-3′	Complementary 3′-UTR sequence, 337 nt after the opal stop codon in Exon 30
γEx30_Rev′	5′-GTC​CTG​CAA​AAG​GAG​ACA​ACG-3′	Complementary to 3′-UTR sequence, 504 nt after the opal stop codon in Exon 30
ζEx1_Fwd	5′-CTG​CGA​GCG​CTC​AGA​TCC-3′	Corresponds to 5′-UTR sequence, 308 nt before the start codon in Exon 1
ζEx1_Fwd′	5′-GAC​AGC​GTC​CCG​CCT​GA-3′	Corresponds to 5′-UTR sequence, 263 nt before the start codon in Exon 1
ζEx12_Rev	5′-CTC​AAT​CAT​GTA​AGC​ATG​TTC​TGA​GAG​ACA​G-3′	Complementary to 3′-UTR sequence, 1,900 nt after the amber stop codon in Exon 12
ζEx12_Rev′	5′-TGT​GCA​CAT​GGG​AAG​TGT​CT-3′	Complementary to 3′-UTR sequence, 463 nt after the amber stop codon in Exon 12
ζEx12a-13_Fwd	5′-GTT​CAG​AGG​CAG​AGG​CCA​GTA​ATA​G-3′	Spans the exon 12“a”/13 splice boundary
ζ12b-13_Fwd	5′-CTC​AAC​CAG​TAT​ACA​ATG​AGG​CCA​GTA​ATA​G-3′	Spans the exon 12“b”/13 splice boundary
ζ20-21a_Rev	5′-CCT​TTC​TGA​GAG​CCC​TTC​TTT​AAC​TTT​G-3′	Spans the exon 20/21“a” splice boundary
ζEx20-21b_Rev	5′- CCC​TGG​AAG​AGT​TCA​GAC​AGC​TTC-3′	Spans the exon 20/21“b” splice boundary
ζEx30_Rev	5′-CAT​GGA​GAC​ACC​AGA​GCA​GTA-3′	Complementary to 3′-UTR sequence, 503 nt after the ochre stop codon in Exon 30
ζEx30_Rev′	5′-AGA​CAA​TGC​ATG​GAT​GAG​GGA​T-3′	Complementary to 3′-UTR sequence, 41 nt after the ochre stop codon in Exon 30

##### RPTPγ primers

We designed two primer sets specifically for *Ptprg* transcripts: one to amplify long transcripts and another to amplify short transcripts. The first (“long”) γEx1_Fwd and γEx30_Rev primer pair amplifies all possible *Ptprg* transcripts that initiate with Exon 1 and terminate with Exon 30, which includes *Ptprg*-V1 (NM_008981) and the hypothetical *Ptprg*-X1 assembly (XM_006517956). The second (“short”) γEx14a_Fwd and γEx30_Rev′ primer pair amplifies the *Ptprg*-V2 transcript (NM_001347593), which initiates in Exon 14a and terminates in Exon 30, 146 nt 3′ to the termination of the previous reverse primer (i.e., γEx30_Rev). We clone the purified PCR bands into the pCR-Blunt plasmid using the Zero Blunt PCR Cloning Kit as described above.

##### RPTPζ primers

Determining the presence of different *Ptprz1* transcripts is more challenging than is for *Ptprg* (which has one validated^1^ short transcript, one validated long transcript, and one hypothetical long transcript; see previous paragraph) for three reasons: (a) *Ptprz1* has as many as nine assemblies, four validated and five hypothetical; (b) *Ptprz1* has multiple “short” (<5.4 kb), one “intermediate” (5.4–8.0 kb), and multiple “long” (>8.0 kb) *Ptprz1* transcripts; and (c) the differences that arise from alternate utilization of three of the cassettes (exon 16, which may be spliced in or out; exon 21“a” or 21“b”^2^) are so small that the alternative splicing yields multiple full-length transcripts that are difficult to distinguish in a single PCR on the basis of size alone. Therefore, we designed sets of nested primer pairs (Table 2) to amplify cDNA fragments from at most two possible transcripts per PCR and then verified the identity of these transcripts through cloning the cDNA and sequencing. In both Table 2 and the lists below, “V” designates previously verified murine *Ptprz1* variants, whereas “X” designates variants previously designated as hypothetical. As described above, we cloned PCR bands for all short, intermediate, and long *Ptprz1* transcripts into pCR-Blunt for sequencing.

**TABLE 2:** Primer-pair combinations for the second round *Ptprz1* variant-specific PCR amplicons. The primers in the first round of PCR were always ζEx1_Fwd and ζEx30_Rev, as summarized in Table 1. In the first column, the solidus flanks two variants that the primer set could amplify. In the last column, the solidus flanks the base-pair lengths of the respective variants.

*Ptprz1* variant	Primers				
	ζ12b-13_Fwd	ζEx12a-13_Fwd	ζ20-21a_Rev	ζEx20-21b_Rev	Amplicon size (bp)
V4/V5	✓		✓		826/805
X1/X2		✓		✓	826/806
V3/X3		✓	✓		820/799
X4/X5	✓			✓	832/811

**V1:** To amplify the lone intermediate-length *Ptprz1*-V1 transcript, we used, in the round-one PCR, ζEx1_Fwd together with the ζEx12_Rev primer. We then used the purified amplicon from round one as template in a second, nested PCR using the ζEx1_Fwd′ and ζEx12_Rev**′** primer pair.

**Other variants:** To amplify all possible “short” and “long” *Ptprz1* full-transcripts that commence with exon 1 and end with exon 30, in round-one PCR, we used the primers ζEx1_Fwd and ζEx30_Rev. For the second round, we used the amplicons from round one as templates, together with the ζEx1_Fwd′ and ζEx30_Rev′ primer pair.

To identify the presence of all other alternative *Ptprz1* cassettes from transcripts that commence with exon 1 and end with exon 30, we used first-round PCR amplicons amplified from PCRs primed with ζEx1_Fwd and ζEx30_Rev as the cDNA template in second-round nested PCRs. These second-round PCRs were primed with new sets of “diagnostic” nested primers (also summarized in Table 1) that produce shorter, variant-specific amplicons. We outline the rationale for the designs of these novel second-round primers in the following list:

**V3/X3:** We designed the ζEx12a-13_Fwd and ζEx20-21a_Rev primer pair that will amplify an 820-bp fragment of *Prprz1*-V3 and/or a 799-bp fragment of *Prprz1*-X3 cDNA if the transcripts are present in TRNA.

**V4/5:** The ζ12b-13_Fwd and ζ20-21a_Rev primer pair amplifies an 826-bp fragment of *Prprz1*-V4 and/or 805-bp fragment of *Prprz1*-V5 if these transcripts are present in TRNA.

**X1/X2:** We designed the ζEx12a-13_Fwd and ζEx20-21b_Rev primer pair to amplify an 827-bp fragment of *Prprz1*-X1 and/or 806-bp fragment of *Prprz1*-X2 if mRNAs are present in the TRNA sample.

**X4/5:** The ζ12b-13_Fwd and ζEx20-21b primer pair amplify an 832-bp fragment of *Ptprz1*-X4 and/or 811 bp fragment of *Ptprz1*-X5 if the transcripts are present in the TRNA sample.

### Antibodies

**RPTPγ**. An RPTPγ chicken IgY antibody raised against the murine RPTPγ sequence ^390^CZNEDEKEKTFTKDSDKDLK^407^ (Mafficini et al., 2007)—Figure 2B, variant V1—was a generous gift from Claudio Sorio (University of Verona, Italy) and used in both ICC and IHC experiments at 2.5 μg ml^–1^ working concentration, diluted from the affinity-purified antibody stock (1.2 mg ml^–1^).

**RPTPζ.** We developed a new rabbit polyclonal antibody (GenScript, Piscataway, NJ) against an extracellular epitope between the FNIII and TM domains of murine RPTPζ (anti-RPTPζ vs ^690^QIDESRETTESFSP^703^; Figure 6B, magenta arrowhead, common to all variants). The antibody was affinity purified, and the titer was determined to be >1:512,000 by indirect ELISA. Aliquots of affinity-purified anti-RPTPζ (stock concentration, 0.794 mg ml^–1^) in phosphate-buffered saline (PBS) with 0.02% sodium azide (pH 7.40) were stored at −80°C. The working dilution was 1:1,000 from the affinity-purified antibody stock solution for cell cultures and 1:500 for tissue sections. We validated the anti-RPTPζ antibody specificity on RPTPζ^−/−^ mixed neuron–astrocyte HC cultures and brain slices.

**Other primary antibodies.** To identify CNS neurons in cultures or tissue sections, we used microtubule-associated protein 2 (MAP2) antibody (mouse IgG1 1:500 dilution; cat# MAB3418, Sigma-Aldrich). To identify astrocytes either in culture or tissue, we stained these with the GFAP antibody (GFAP, mouse IgG1 1:400 dilution; cat# G3893, Sigma-Aldrich).

**Secondary antibodies.** We applied 1 μg ml^–1^ of secondary antibodies conjugated to Alexa Fluor 633 to detect the binding of the primary antibodies against RPTPγ (cat# A-21103, Thermo Fisher Scientific, goat anti-chicken) or RPTPζ (cat# A-21070, Thermo Fisher Scientific, goat anti-rabbit). We detected the anti-MAP2 or anti-GFAP binding by incubating samples with 1 μg ml^–1^ of goat anti-mouse secondary antibody conjugated to Alexa Fluor 488 (cat# A-28175, Thermo Fisher Scientific). We counterstained cell nuclei with 4,6-diamidino-2-phenylindole (DAPI, 300 μM working concentration, diluted from a 30 mM stock dissolved in dimethylformamide).

### Immunocytochemistry

We aspirated the media from mixed neuron–astrocyte HC cultures and washed 3× with ice-cold PBS. We then fixed the cultures by incubating them for 20 min in 4% paraformaldehyde (PFA, cat# 15710, Electron Microscopy Sciences) in PBS. We then permeabilized the cells by incubating the samples in PBS + 0.05% Tween 20 (PBST) for 10 min. For the primary antibodies raised in mouse or rabbit, we blocked non-specific antibody binding by incubating the fixed and permeabilized cells in 5% normal goat serum (NGS) in PBS for 30 min at room temperature. For samples stained with the chicken RPTPγ primary antibody, we blocked the fixed and permeabilized cells with 1% bovine serum albumin (cat #9048-46-8, Sigma) in PBS for 30 min at room temperature to minimize non-specific binding. We then incubated the fixed, permeabilized, and blocked samples with primary antibodies overnight at 4°C. Following the overnight primary antibody incubation, we washed the cells 3× with PBST and then again blocked with either 5% NGS or 1% BSA (for RPTPγ stained samples) in PBS for 60 min, all at room temperature. Subsequently, we incubated the cells for 60 min at room temperature with the appropriate fluorescently tagged secondary antibodies (see above). Finally, we washed the samples with PBST 3× and during the final wash, counterstained them with the nuclear marker DAPI. We mounted the stained coverslips in VECTASHIELD Antifade Mounting Medium (cat# H-1000-10, VectorLabs) on microscope slides, sealed the coverslip edges with clear nail polish, and left them to cure in the dark for at least 1 h before imaging or before storing the slides at 4°C until imaging.

### Immunohistochemistry

In the case of newborns, we decapitated P0–P2 pups, immediately excised the brains, and identified and isolated the hippocampi, which we placed into 4% PFA in PBS overnight at 4°C. The following day, the brains were switched to 2% PFA in PBS for 24 h at 4°C. In the case of adults, we placed mice under deep isoflurane-induced anesthesia and then transcardially perfused each animal, first with heparinized normal saline (0.9% NaCl in H_2_O with 10 units heparin per milliliter; cat# H0777, Sigma) to flush out the blood and then with 4% PFA in PBS. We immediately excised the brains and placed them into 2% PFA overnight at 4°C.

For fixed pup and adult brains, we performed sucrose exchange before embedding the whole brains in O.C.T. Compound (cat# 4586, Scigen), freezing, and cryosectioning. We cut and mounted 5-µm-thick sagittal brain sections on Superfrost Microscope Slides (cat# 12-550-15, Fisher Scientific) and stored them at −20°C until ready for use.

Prior to staining, we performed antigen retrieval by microwaving the sections in 2 mM citric acid/8 mM sodium citrate buffer, pH 6.0, first for 4 min at high power and then for 10 min at 50% power (in a 1200-W microwave). After microwaving, we placed the slides in a beaker, packed the beaker in ice, and allowed the sections to cool to room temperature while still keeping them submerged in citrate buffer. After cooling, we washed slides 3× 5 min with Tris-buffered saline + 0.05% Tween 20 (TBS-T), and then air-dried for 10 min. After delineating each section using a PAP Pen (cat# 195505, Research Products International Corp.), we rehydrated the slides 3× for 5 min with TBS-T and then blocked with 1% BSA, 4 nM NaN_3_ in TBS-T for 30 min to minimize non-specific binding. Primary antibodies were diluted from stock to their working concentrations in the appropriate blocking buffer (mouse monoclonal and rabbit polyclonal primary antibodies in 5% NGS in TBS, and chicken primary antibody in 1% BSA in TBS; see Antibodies, Secondary antibodies) and incubated overnight at 4°C. The next day, we performed a 5-min wash in TBS-T + 1% BSA, followed by a high-salt TBS wash (2.5% NaCl), and then another 5-min wash in TBS-T + 1% BSA. We diluted secondary antibodies in the appropriate blocking buffer as described above for the cultured cell staining and incubated in the dark for 2 h at room temperature. We next performed a 5-min wash in TBS-T + 1% BSA, followed by a high-salt TBS wash (2.5% NaCl), and then another 5-min wash in TBS. In the subsequent step, we then performed auto-fluorescence quenching using the TrueVIEW Autofluorescence Quenching Kit (cat# SP-8400-15, VectorLabs, Newark, CA) according to the manufacturer’s instructions. We performed a final 5-min wash in TBS before we mounted the stained sections in VECTASHIELD Antifade Mounting Medium. Finally, we sealed the coverslip edges with clear nail polish and left them to cure in the dark for at least 1 h before either imaging or storing the slides (at 4°C) for imaging.

### Confocal microscopy

We acquired images using an FV3000 (IX83) laser-scanning confocal microscope equipped with a super-corrected PLAPON60×OSC2, 1.4 numerical aperture oil-immersion objective lens. For co-labeling studies, we scanned each laser line in an individual channel to minimize bleed-through, which was negligible. We acquired images utilizing the full dynamic range of the acquisition system, that is, setting laser intensity, photomultiplier tube voltage, and offset based on the laser wavelength and intensity of the specimen fluorophores. We sampled images at 12 bits/pixel with a dwell time of 10.0 μs/pixel.

## Results

### RPTPγ

#### Identification of a novel RPTPγ variant in mouse hippocampus

**FIGURE 1:**
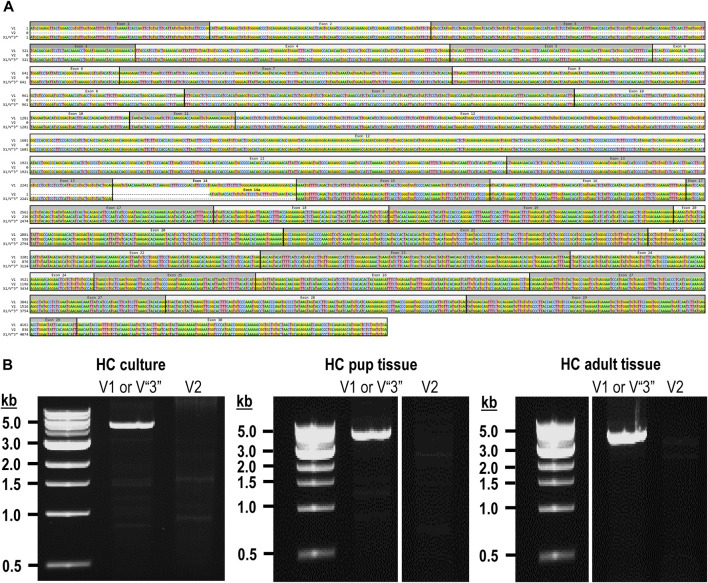
Nucleotide alignment of RPTPγ transcript variant ORFs. **(A)** Multiple sequence alignment of the ORF nucleotide sequences for the mouse variants RPTPγ-V1 (NM_008981), RPTPγ-V2 (NM_001347593), and RPTPγ-V“3” (formerly XM_006517956). We delineate exon boundaries with black boxes and provide the exon numbers above the nucleotide sequences (odd exon numbers on a gray background, even numbers on a white background). Note that alternative splicing yields exon 14 in V1 (white background), the completely different exon 14a in V2 (yellow background), and the exclusion of both exons 14 and 14a from V“3”. **(B)** Bands amplified from RT-PCRs using gene-specific primers designed to amplify full-length V1 or V2 from TRNA isolated from mixed neuron–astrocyte HC cultures (left), HC tissue from P0–P2 pups (center), and adult mouse HC tissue (right). The lane labels describe the primer set (Table 1) used to perform each PCR. Clearly visible in all preparations is the ∼5-kb band, which we isolated and cloned. The band contained cDNAs for both V1 and V“3”. In none of the three preparations did the V2 gene-specific primers produce a readily visible product of the predicted 2,598-bp size. The “HC pup tissue” gel image is spliced (marked by the vertical white line) to remove one lane that were identical replicates of the displayed V1 or V“3” lane. The “HC adult tissue” panel is from the same gel image as the “HC pup tissue” image. This “HC adult tissue” image is spliced (marked by the vertical white line) to remove four lanes that represented “HC pup tissue” data.

The mouse *Ptprg* gene (Gene ID: 19270) comprises 31 exons on chromosome 14. When we commenced the present investigation, the NCBI RefSeq database (Pruitt et al., 2012) contained two murine splice variants (i.e., V1 and V2). The variant 1 (*Ptprg*-V1, [NM_008981]) transcript is encoded by exons 1–30 (Figure 1A).

The variant 2 (*Ptprg*-V2, [NM_001347593]) transcript differs from V1 in that it does not include exons 1–13 but rather originates with the alternate exon 14a, which contains an alternative in-frame initiator methionine and then assembles with exons 15–30 to generate a transcript that lacks the coding regions for the extracellular and TM domains of V1 (Figure 1A).

In the present study, we designed gene-specific primers (GSPs; Table 1) for use in RT-PCR cloning experiments to determine which RPTPγ variants are expressed in mixed neuron–astrocyte HC cultures, P0–P2 pup hippocampi, and adult HC tissue. We designed the γEx1_Fwd and γEx30_Rev GSPs to amplify the RNA encoding the known V1 variant and γEx14a_Fwd and γEx30_Rev′ to detect the known V2. From all three preparations, γEx1_Fwd and γEx30_Rev GSPs amplified ∼5,000 bp bands, which we cloned and sequenced. Each band actually represents two transcripts, one encoding the expected V1 variant and the other encoding a novel variant that we propose to designate as V3. Throughout the article, we use double quotation marks to enclose proposed variant numbers (i.e., those not yet formalized by the NCBI or other bodies). Thus, we write proposed V3 as V“3”.

##### V1 transcripts

From mixed neuron–astrocyte HC cultures, we obtained 10 colonies, 40% of which contained the complete 4,329-bp open reading frame (ORF) for *Ptprg*-V1 (Figure 1B). We submitted three of these four clones to GenBank, which assigned the accession numbers [OR710276], [OR710277], and [OR710278]. From pup and adult HC tissue, we obtained 10 colonies each, 30% of which contain the same complete 4,329-bp ORF for *Ptprg*-V1. For both pup and adult, we submitted two samples for pup and three for the adults of these three clones to GenBank. GenBank assigned accession numbers [OR710279] and [OR710280] for the pup, and [OR710281], [OR710282], and [OR710283] for the adults.

##### Novel V“3” transcripts

In addition to the 40% or 30% of the PCR cloning colonies that yielded *Ptprg*-V1 cDNA (see V1 transcripts section), we found that the remaining colonies represent the hypothetical murine assembly [XM_006517956]. Specifically, 60% of the colonies from mixed neuron–astrocyte HC cultures (five submitted, yielding GenBank accession numbers [OR900076]–[OR900080]) and 70% of the PCR cloning colonies from pup ([OR900081]–[OR900088]) or adult HC tissue ([OR900089]–[OR900093]; Figure 1B). This novel transcript lacks exon 14 (representing 29 amino acids), which encodes the intracellular region immediately after the TM domain in *Ptprg*-V1.

Interestingly, in a single pup P0–P2 culture clone (i.e., [OR900082]), sequencing on both cDNA strands revealed the existence of a single G→A nucleotide substitution, 584 nt into the *Ptprg* ORF. In [OR900082], this substitution changes the ^583^AGA^585^ ORF codon to ^583^AAA^585^, resulting in an R195K amino acid change in the translated RPTPγ protein. This conservative substitution, located on the external surface of the CALD, is the single amino acid linking the S186-N194 α-helix and the I197-S207 β-strand (Bouyain and Watkins, 2010). As a point of reference, R195K is located within the CALD, 20 downstream from residue Q175, which is at the position equivalent to one of the three Zn^2+^-coordinating histidines in active CAs.

Note that we did not amplify or clone cDNA corresponding to mouse *Ptprg*-V2 from any of our three mouse preparations (Figure 1B).

**FIGURE 2:**
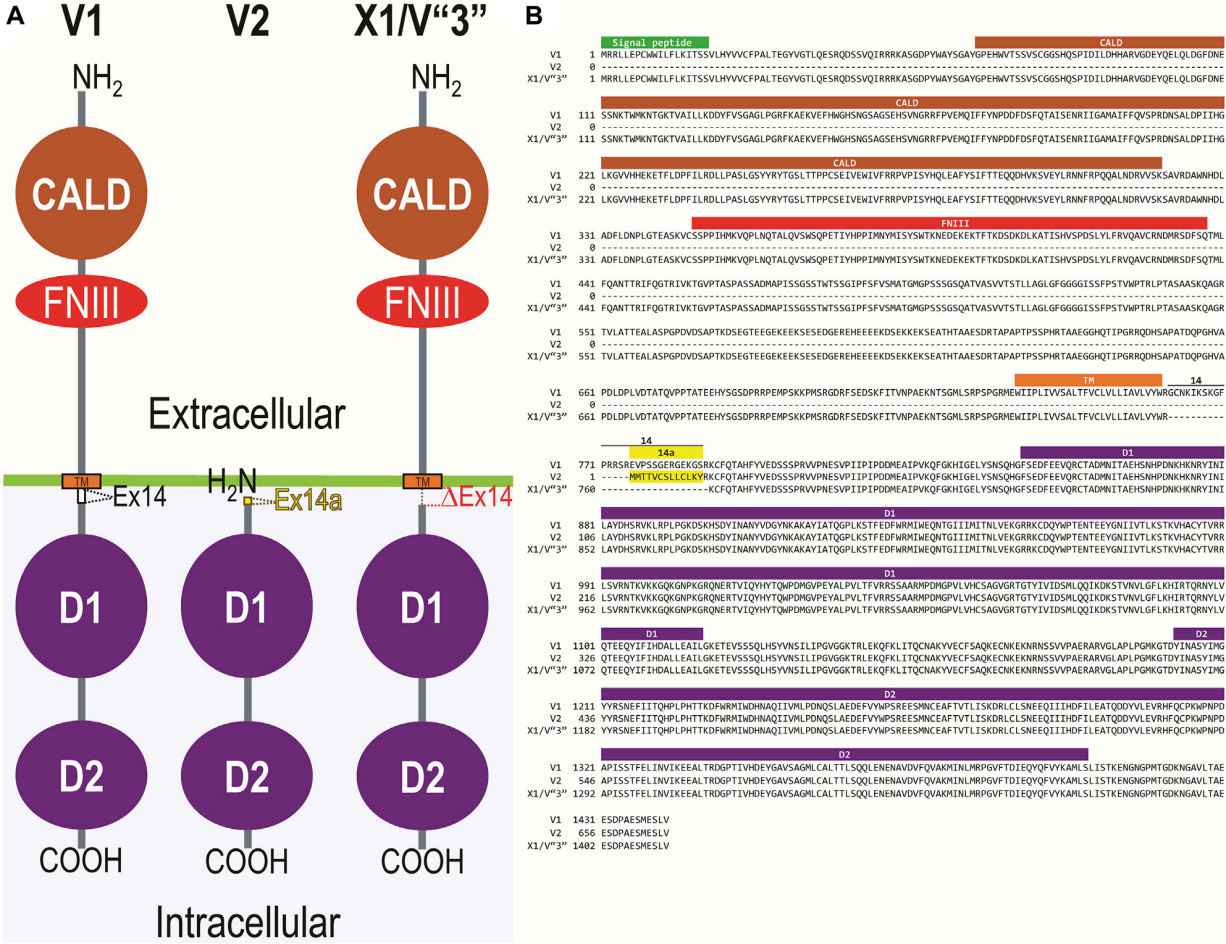
Functional domains and alignment of mouse RPTPγ protein variants. **(A)** Schematic view of the functional domains in mouse RPTPγ protein variants. V1 and V“3” are transmembrane variants that possess extracellular CALD and FNIII domains. RPTPγ-V1 and RPTPγ-V“3” are distinguished by the inclusion, in V1, of a 29-amino acid intracellular cassette (see white box labeled “Ex14”; encoded by exon 14 as shown in Figure 1), immediately after the TM domain (orange box). RPTPγ-V“3” lacks the 29 amino acids encoded by exon 14 in V1 (see gap labeled “ΔEx14” in red). Because the translation of the *Ptprg*-V2 transcript begins with the methionine in exon 14a, RPTPγ-V2 lacks all of the amino acids between the N-termini and transmembrane domains of V1 and V“3” and is thus a cytoplasmic variant that possesses only the 13 amino acids encoded by exon 14a (shown as a yellow box) through the C-terminus, which includes the D1 and D2 domains. The vertical height of each domain is proportional to the number of amino acid resides comprising each domain. **(B)** The amino acid alignment of the three mouse RPTPγ variants. RPTPγ-V1 [NP_033007], RPTPγ-V2 [NP_001334522], and RPTPγ-V“3” (that we proposed becomes the validated form of [XP_00651801]). We highlight the functional domains above each row with shading colors that match the domains in **(A)**. We also highlight the alternative 29-amino acid sequence encoded by exon 14 (black text above alignment) or the alternative 13-amino acid sequence encoded by exon 14a (yellow highlighted text).

Figure 2A displays a schematic representation of the topology and structural domains of the three translated RPTPγ variants. RPTPγ-V1 [NP_033007] and RPTPγ-V“3” (formerly hypothetical [XP_006518019]) both possess extracellular CALD and FNIII domains, followed by a single TM domain, and the D1 and D2 phosphatase domains. They differ only by the omission of 29 juxta-membrane amino acids that, in RPTPγ-V“3”, are encoded by exon 14 (Figure 1A). In Figure 2A, we represent this omission by the dashed line. RPTPγ-V2 [NP_001334522]—undetected in the present study—lacks the CALD, FNIII, and transmembrane domains and therefore is an exclusively intracellular variant.

Figure 2B displays the amino acid sequence alignment for the three RPTPγ variants. The RPTPγ-V“3” protein corresponds to the protein previously only reported by NCBI RefSeq as a hypothetical mRNA XM_006517956 and the hypothetical translation product [XP_006518019].

#### RPTPγ protein expression in HC neurons and astrocytes in mixed culture, pup, and adult tissue

##### Mixed neuron–astrocyte HC cultures

**FIGURE 3:**
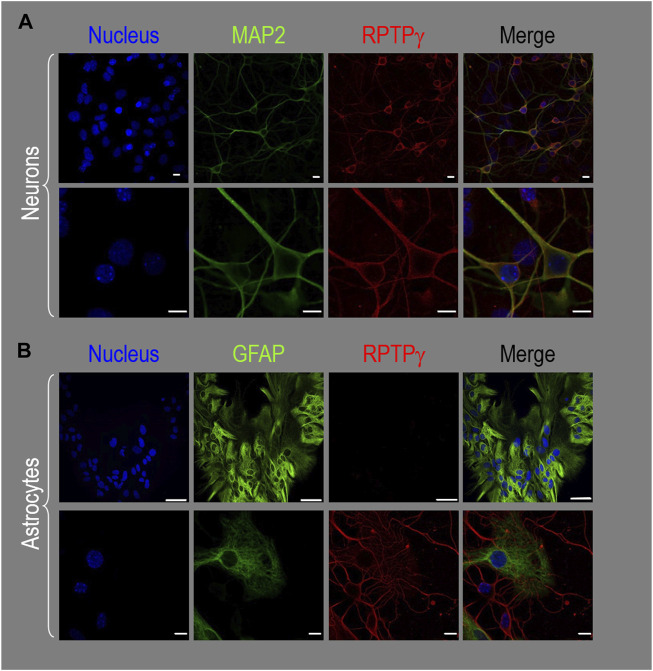
RPTPγ protein expression in HC neurons and astrocytes, in mixed cultures from WT mice. **(A)** Neurons in representative low-magnification (top row) and high-magnification (bottom row) images of mixed neuron–astrocyte HC cultures. The columns of panels (from the left) show DAPI staining (blue) of cell nuclei, MAP2 staining (green) to identify neurons, RPTPγ staining (red), and the merge. Note that both the somata and processes of the green MAP2-positive cells (i.e., neurons) stain for RPTPγ (red). **(B)** Astrocytes in representative low-magnification (top row) and high-magnification (bottom row) images emphasizing astrocytes in mixed neuron–astrocyte HC cultures. The protocol and presentation are the same as in **(A)** except that here we used GFAP staining (green) to identify astrocytes. Note that the green GFAP-positive cells (i.e., astrocytes) lack the red RPTPγ staining, which we observed only in adjacent neuronal somata and processes at high magnification (bottom row). Scale bars in all panels represent 10 μm.

About 14 days after initiating cultures from WT P0–P2 mouse pups, we stained the primary neuron–astrocyte HC cultures with DAPI (Figure 3A, blue in leftmost column), a MAP2 mouse monoclonal antibody (green) to identify the neurons, and a RPTPγ chicken IgY antibody (red; see Mafficini et al., 2007). The rightmost column shows the merged images. We determine that RPTPγ (Figure 3A, red) is localized throughout the neuronal soma and major neuronal processes. When we stained parallel cultures, not with an MAP2 antibody but with a GFAP mouse monoclonal antibody to identify the astrocytes (Figure 3B, green), RPTPγ-stained projections (red) from adjacent neurons are clearly visible surrounding the astrocytes. However, the staining never colocalizes within the GFAP-positive cells (i.e., astrocytes).

##### P0–P2 pup HC tissue

**FIGURE 4:**
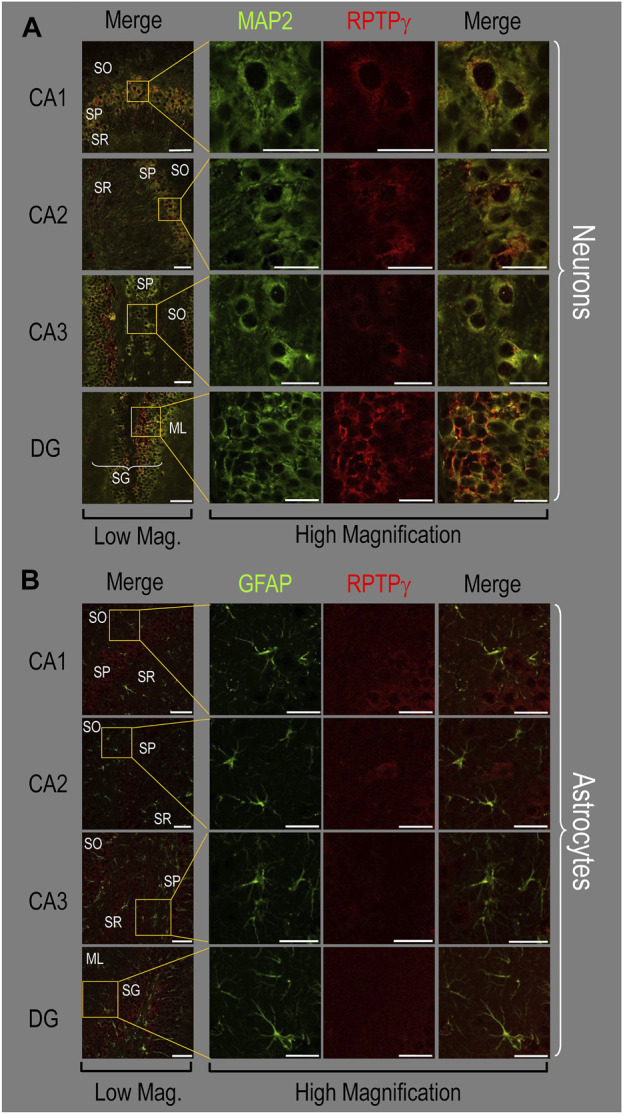
RPTPγ protein expression in P0–P2 pup HC tissue from WT mice. **(A)** Neurons in representative examples of low- and high-magnification images of RPTPγ immunostained tissue. The columns of panels (from the left) show (a) low-magnification merged images of MAP2 staining (green; individual images not shown) and RPTPγ staining (red; individual images not shown), and then three columns of high-magnification zoomed-in images representing (b) MAP staining (green), (c) RPTPγ staining (red), and (d) the merge. The rows of panels (from the top) show images of the CA1, CA2, CA3, and DG regions. In the low-magnification images, the gold squares delineate the ROIs into which we zoomed-in the three higher-resolution panels to the right. Within the CA regions, we annotate the SO, SP, and SR. Within the DG, we annotate the ML and SG. Note that the neuronal MAP2 marker (green) colocalizes with RPTPγ (red), as indicated in yellow in the merged images, acquired at both low and high magnification. **(B)** Astrocytes in representative examples of low- and high-magnification images of RPTPγ immunostained tissue. The protocol and presentation are the same as in **(A)**, except that here we used GFAP staining (green) to identify astrocytes. Note that the green GFAP-positive cells and processes (i.e., astrocytes) in the CA1-3 regions and DG lack the red RPTPγ staining. The magnified ROIs further highlight that only the neuropil surrounding GFAP-positive cells (i.e., presumably neurons) faintly stains for RPTPγ. Scale bars in all panels represent 10 μm.

Because we derived the mixed neuron–astrocyte HC cultures from P0–P2 pups, we also studied RPTPγ expression in native P0–P2 HC tissue. We examined stained sections from four regions of P0–P2 pup hippocampus: cornu ammonis 1 (CA1), CA2, CA3, and dentate gyrus (DG). In each row of Figure 4, the leftmost panel is at relatively low magnification, whereas the panels to the right are high-magnification images from selected regions of interest (ROIs). We observed that MAP2-stained neurons (Figure 4A, green) in all four HC regions also stained for RPTPγ (red). Throughout the CA regions, the brightest RPTPγ staining was in the SP and became more diffuse in the adjacent stratum oriens (SO) and stratum radiatum (SR). Within the DG, RPTPγ staining was concentrated in the stratum granulosum (SG) but extended more diffusely into the neuropil of the adjacent molecular layer (ML).

In parallel sections, we stained astrocytes with anti-GFAP (Figure 4B, green) and observed no colocalization of RPTPγ (red) throughout the CA1, CA2, CA3, or DG with GFAP-positive cells (i.e., astrocytes) or processes. Positive RPTPγ staining was only diffusely visible in the surrounding neuropil.

##### Adult HC tissue

**FIGURE 5:**
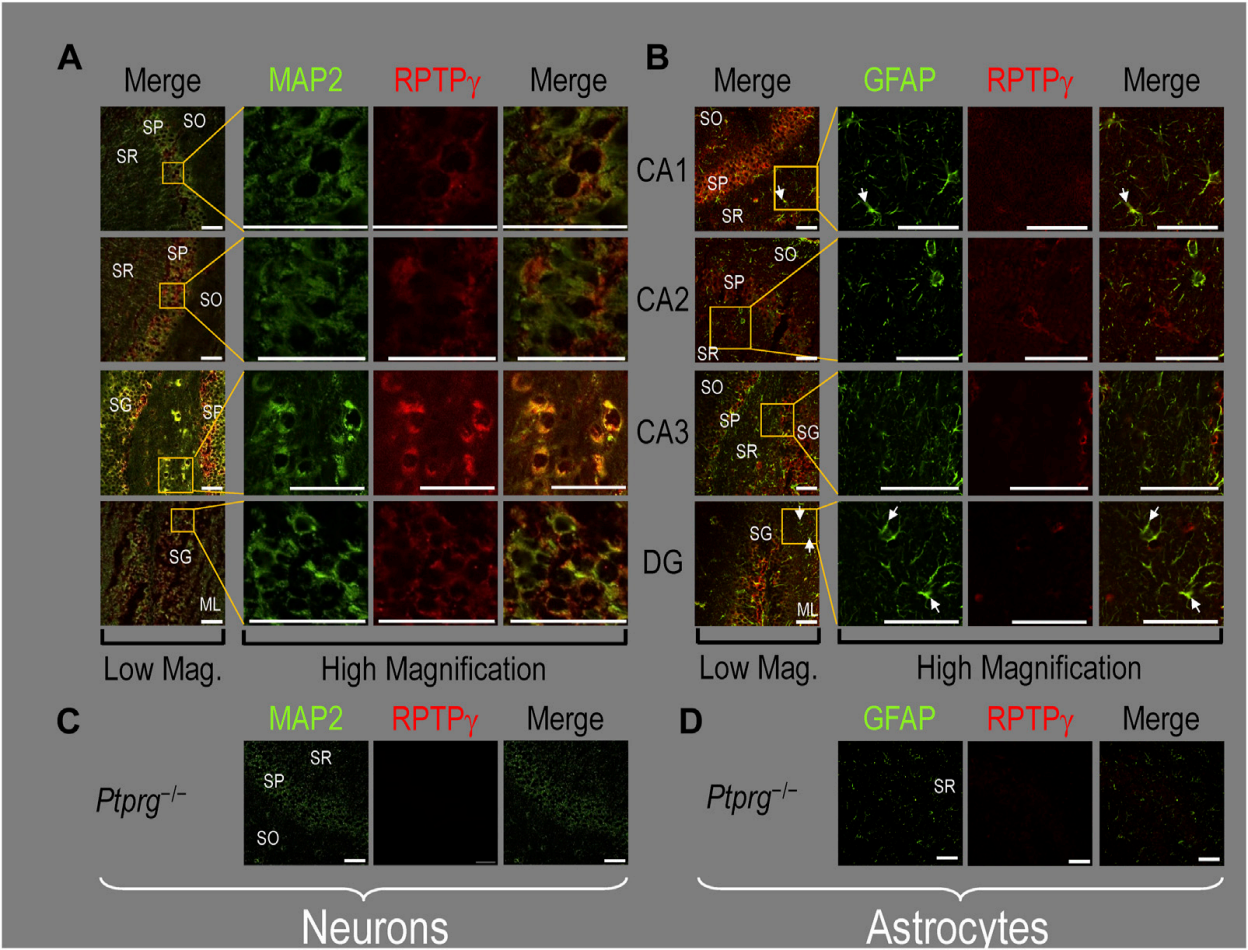
RPTPγ protein expression in adult HC tissue from WT and *Ptprg^-/-^* mice. **(A)** Neurons in representative examples of low- and high-magnification images of RPTPγ immunostained tissue from WT mice. The columns of panels (from the left) show **(A)** low-magnification merged images of MAP2 staining (green; individual images not shown) and RPTPγ staining (red; individual images not shown), and then three columns of high-magnification zoomed-in images representing **(B)** MAP staining (green), **(C)** RPTPγ staining (red), and **(D)** the merge. The rows of panels (from the top) show images of the CA1, CA2, CA3, and DG regions. In the low-magnification images, the gold squares delineate ROIs, into which we zoomed-in the three higher-resolution panels to the right. Within the CA regions, we annotate the SO, SP, and SR. Within the DG, we annotate the ML and SG. Note that the neuronal MAP2 marker (green) colocalizes with RPTPγ (red), as indicated in yellow in the merged images, acquired at both low and high magnifications. **(B)** Astrocytes in representative examples of low- and high-magnification images of RPTPγ immunostained tissue. The protocol and presentation are the same as in **(A)**, except that here we used GFAP staining (green) to identify astrocytes. Note that the green GFAP-positive cells and processes (astrocytes; e.g., see white arrows) in the CA1-3 regions and DG lack the red RPTPγ staining that appears only in neighboring neuronal processes. The magnified ROIs further highlight that only the neuropil surrounding GFAP-positive cells (i.e., presumably neurons) faintly stains for RPTP. **(C)** Neurons in representative examples of low-magnification images of RPTPγ immunostained tissue from *Ptprg^-/-^* mice. Here, we validate the chicken IgY RPTPγ antibody. The protocol and presentation are the same as in **(A)**. **(D)** Astrocytes in representative examples of low-magnification images of RPTPγ immunostained tissue from *Ptprg^-/-^* mice. The protocol and presentation are the same as in **(B)**. Scale bars in all panels represent 10 μm.

Finally, following our approach for cultures and pups, we examined RPTPγ expression in native adult (8- to 12-week-old animals) HC tissue. We found expression patterns for RPTPγ (Figure 5A) that are nearly identical to what we observed in the P0–P2 pup tissue. In particular, RPTPγ colocalizes strongly with MAP2-positive cells (i.e., neurons) in the CA1–3 SP HC regions. In the DG, RPTPγ staining localizes to the soma of the granule cell within the SG.

In adult mouse HC tissue stained for GFAP (Figure 5B, green), but not for MAP2, we consistently observed GFAP-positive cells (i.e., astrocytes) to be devoid of RPTPγ staining (red). Rarely, and especially at low magnification, we observe yellow pixels in a merged image (i.e., GFAP from astrocytes plus RPTPγ). The yellow could reflect (a) a neuron and an astrocyte in the same optical section or (b) the rare astrocyte that expresses RPTPγ (see Discussion). Nevertheless, the dominant pattern is that GFAP-positive cells are RPTPγ negative. GFAP-positive astrocyte soma and, in some cases, the early branches of their processes are most clearly visible in the CA1–3 SR in the high-magnification ROIs of Figure 5B, where GFAP and RPTPγ do not appear to colocalize within individual cells or cell processes.

To validate the RPTPγ chicken IgY antibody, we immunostained adult *Ptprg* ^−/−^ HC sections following the same protocols as used in Figures 4A, B, 5A, B. After counterstaining with either MAP2 (Figure 5C) or GFAP (Figure 5D), we did not observe any non-specific signal from the anti-RPTPγ chicken IgY.

### RPTPζ

#### Identification of novel RPTPζ variants in mouse hippocampus

##### Analysis of Ptprz1 transcripts, as known at the initiation of the present study

The mouse *Ptprz1* gene (Gene ID: 19283) contains 30 exons on chromosome 6. When the present investigation commenced, the NCBI RefSeq database contained four validated murine splice variants (i.e., V1, V3, V4, and V5) that had also been described by Fujikawa et al. (2017) and five hypothetical transcript assemblies (i.e., X1 through X5).

Here, we define exon 12 as the full-length exon. We propose the names 12“a” and 12“b” for the shorter alternative exons that arise from splicing at one of two splice sites within exon 12, such that 12“a” is entirely within 12 and 12“b” is entirely within 12“a”.

**FIGURE 6:**
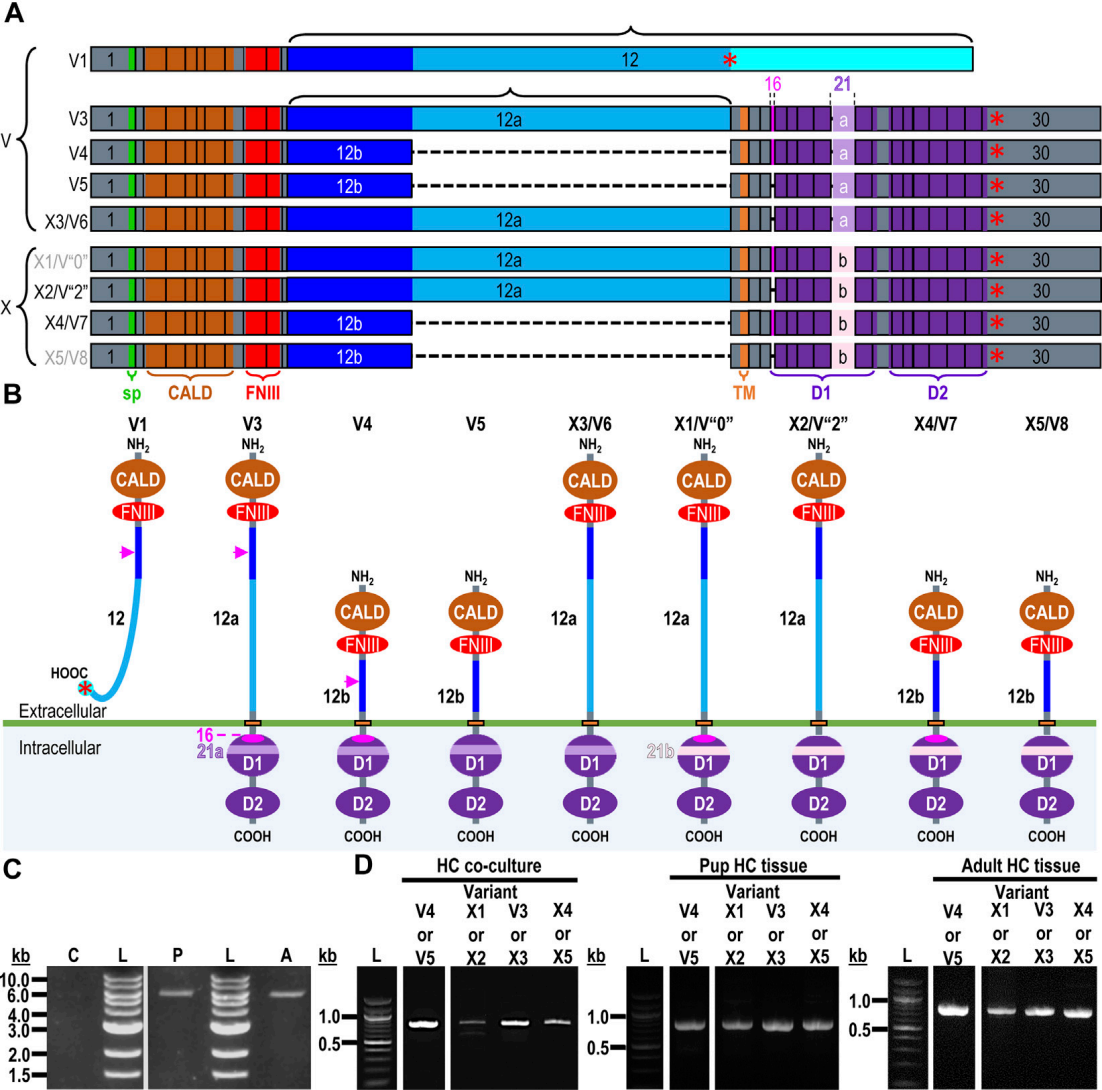
RPTPζ variants. **(A)** Mouse *Ptprz1* transcript variants. Each box represents an exon in the mature transcript. We draw the exon boxes to scale, with the longest transcript, *Ptprz1*-X1, being 8,268-nt long. Exon 1 and Exon 30 are annotated, while for clarity, exons 2–11 and 13–15 (that are included in all variants) and exons 17–20 and 22–29 (that are included in all variants except *Ptprz1*-V1) are not numbered. We annotate only with the numbers for the alternatively spliced or alternatively included *Ptprz1* variants (exon 12, 12“a”, or 12“b”; exon 16; and exon 21“a” or 21“b”). The region encoding the signal peptide (sp) is in green within exon 1. The region encoding the CALD is highlighted in orange within exons 3–8. The FNIII encoding region is in red within exons 9 and 10. The alternatively spliced exons 12, 12“a”, and 12“b” are in blue. The highlighted region within exon 13 encodes the transmembrane domain (TM). The regions encoding the intracellular D1 (within exons 15–23) and D2 (within exons 23–30) phosphatase domains are in purple. The violet shading within these regions highlights the inclusion of the alternatively spliced exons 16 and 21“a” or 21“b”. A red asterisk highlights the location of the translation stop codon in each variant. We label each variant according to its NCBI RefSeq validated nomenclature at the time when the present investigation commenced. If applicable, alternative classifications of the hypothetical assemblies are also shown, if, since we commenced the present investigation, it has been updated to a validated/expressed transcript by the NCBI RefSeq or by the results presented in this article. **(B)** A schematic view of the functional domains in mouse RPTPζ variant proteins: RPTPζ-V1 [NP_035349], RPTPζ-V3 [NP_001074775] and RPTPζ-V4 [NP_001297993], RPTPζ-V5 [NP_001348278], RPTPζ-X3/V6 [NP_001389981], RPTPζ-X1 [XP_006505075], RPTPζ-X2/V“2” [XP_006505076], RPTPζ-X4/V7 [NP_001389982], and RPTPζ-X5/V8 [NP_001389983]. The height of each protein and its intracellular domains are drawn to scale, with the longest variant, RPTPζ-X1, being 2,318 amino acids long. The CALD, FNIII, TM, D1, and D2 phosphatase domains are annotated and colored as in **(A)**, which include the expression cassettes encoded by the alternatively spliced or utilized exons 12, 16, and 21. RPTPζ-V1 is an extracellularly secreted variant that lacks a TM domain due to the inclusion of exon 12 in its transcript. The magenta arrowheads indicate the location of the anti-RPTPζ-antibody epitope generated in the present investigation, which is common to all RPTPζ variants but, for clarity, is only annotated on RPTPζ-V1, RPTPζ-V3, or RPTPζ-V4. RPTPζ-V3, RPTPζ-X3/V6, RPTPζ-X1, and RPTPζ-X2/V“2” possess a long extracellular linker between the FNIII and TM domains due to their utilization of exon 12“a”. RPTPζ-V4, RPTPζ-V5, RPTPζ-X4/V7, and RPTPζ-X5/V8 have much shorter FNIII-TM domain linker due to their utilization of exon 12“b”. The remaining sites of variation are all within the D1 phosphatase domain due to the alternative usage of exons 16, 21“a”, and 21“b”. **(C)** Products of RT-PCRs using nested gene-specific primers designed to amplify almost full-length *Ptprz1*-V1 from cDNA transcribed from TRNA isolated from mixed neuron–astrocyte HC cultures (C), P0–P2 pup HC tissue (P), and adult mouse HC tissue (A). We did not amplify a *Ptprz1*-V1-specific band from the mixed neuron–astrocyte HC culture cDNA, but bands are present in both the P0–P2 pup and adult HC tissue lanes. Lanes in which we ran a 1-kb ladder are annotated “L”. **(D)** 799- to 832-bp products of the RT-PCRs using nested GSP primers (Table 2) are designed to amplify cDNA fragments indicative of the *Ptprz1* variants annotated above each lane. The gels are ordered from left to right according to the source of the cDNA template for these reactions: mixed neuron–astrocyte HC cultures (left panel), P0–P2 pup HC tissue (center panel), and adult mouse HC tissue (right panel). The panels within each tissue source group (i.e., HC co-culture, P0–P2 pup HC tissue, or adult HC tissue) are all from the same gel images, but the white vertical lines mark where we cropped unused lanes out of each image. The leftmost lanes containing a 100-bp ladder are annotated “L”.

***Ptprz1*-V1 (Exons 1–12).** Variant 1 (*Ptprz1*-V1, [NM_011219]) is encoded by exons 1–12 (Figure 6A; Table 3). V1 is the only variant to utilize the 5479-nt exon 12 that, due to the presence of the ^5456^AAUAAA^5461^ cleavage and polyadenylation specificity factor binding site, will be cleaved and polyadenylated 18-nt downstream, preventing its splicing to any additional exons. Utilization of exon 12 in *Ptprz1*-V1 results in the ORF continuing only as far as an in-frame amber (TAG) stop codon, located at a position analogous to 3-nt downstream from the exon 12/13 splice site in V3. The remainder of the *Ptprz1*-V1 mRNA, downstream of the amber (TAG) stop codon, consists of a 1,938-nt 3′-UTR. At the protein level, V1 encodes an RPTPζ variant that has 1,211 amino acids between the end of the FNIII domain and the termination of the protein, which lacks transmembrane and phosphatase domains (Figure 6B). Thus, V1 is secreted (S) into the extracellular fluid. Fujikawa et al. (2017) originally designated V1 as PTPRZ-S to reflect the ultimate fate of the protein.

**TABLE 3:** Summary of validated and hypothetical Ptprz1 transcript assemblies at the time when the present study was initiated vs the time of study conclusion. The “long” FNIII-TM linker is 1,232 amino acids in length, and the short FNIII-TM linker is 383 amino acids long. When the 7 amino acids coded by exon 16 are included in the *Ptprz1* transcript, the helix-turn-helix “wedge” segment of the D1 phosphatase domain is “large”. In Δ16 *Ptprz1* transcripts, the D1 wedge segment is small. Abbreviations: Fibronectin type III domains (FNIII), transmembrane domain (TM), the D1 PTPase domain (D1), and the D1 catalytic site (CS). HC neuron–astrocyte cultures (C), P0–P2 pup HC tissue (P), and adult HC tissue (A). *Ptprz1*-V0 and *Ptprz1*-V2 are our proposed designations for newly validated variants. Similarly, 12a, 12b, 21a, and 21b are our proposed designations for alternative exons.

Transcript name at study initiation	NCBI accession # at initiation of present study	Other names for transcript	Updated transcript name	Updated NCBI accession #	Variable exons			Variable peptide segments			mRNA expression		
								FNIII-TM linker	D1 wedge	±6-amino acid nt to D1 CS	C	P	A
*Ptprz1*-V1	NM_011219	*Ptprz1*-S			12	Δ16	Δ21	N/A	N/A	N/A	−	+	+
*Ptprz1*-V3	NM_001081306	*Ptprz1*-A			12a	16	21a	Long	Large	−	+	+	+
*Ptprz1*-V4	NM_001311064	*Ptprz1*-B			12b	16	21a	Short	Large	−	+	+	+
*Ptprz1*-V5	NM_001361349	*Ptprz1*-BΔ*ex16*			12b	Δ16	21a	Short	Small	−	+	+	+
*Ptprz1*-X3	XM_006505014	*Ptprz1*-AΔ*ex16*	*Ptprz1*-V6	NM_001403052	12a	Δ16	21a	Long	Small	−	+	+	+
*Ptprz1*-X1	XM_006505012		*Ptprz1*-V0		12a	16	21b	Long	Large	+	+	+	−
*Ptprz1*-X2	XM_006505013		*Ptprz1*-V2		12a	Δ16	21b	Long	Small	+	+	+	+
*Ptprz1*-X4	XM_006505015		*Ptprz1*-V7	NM_001403053	12b	16	21b	Short	Large	+	+	+	+
*Ptprz1*-X5	XM_006505017		*Ptprz1*-V8	NM_001403054	12b	Δ16	21b	Short	Small	+	−	+	+

**Exon 1–30 variants (*Ptprz1*-V3 to *Ptprz1*-V5 and *Ptprz1*-X1 to *Ptprz1*-X5).** The eight other validated or hypothetical *Ptprz1* transcripts all commence with exon 1 and end with exon 30 (Figure 6A). None of these eight variants includes exon 12. Instead, the source of variation comes from (a) utilization of one of the two other exon-12 sites that splice to exon 13, yielding shorter exon 12 variants (i.e., 12“a” or 12“b”); (b) inclusion vs omission of exon 16; and (c) utilization of one of two possible exon-21 alternatives (i.e., 21“a” or 21“b”; Table 3). Below, we outline the alternative cassette usage by each of the eight exon 1–30 variants.

***Ptprz1*-V3 [NM_001081306]—Exon 12“a”, exon 16, exon 21“a”.** (a) If the splice site 3,547-nt into exon 12 is utilized, the result is alternative exon 12“a”, and this splices to exon 13 (Figure 6A; Table 3). At the protein level, V3 has 1,232 amino acids between the end of the FNIII domain and the start of the TM domain (Figure 6B; Table 3). (b) The middle portion of the *Ptprz1*-V3 transcript, beginning at exon 13, includes exon 16. (c) The last portion of the *Ptprz1*-V3 transcript, beginning at exon 17, includes exon 21“a” rather than 21“b” (Figure 6A; Table 3). The full *Ptprz1*-V3 transcript, which Fujikawa et al. (2017) named *Ptprz*-A, comprises 8,018 nt and encodes a 2,305-amino acid protein.

***Ptprz1*-V4 [NM_001311064]—Exon 12“b”, exon 16, exon 21“a”.** If the splice site at the position 1,000-nt into exon 12 is utilized, the result is exon 12“b”. This is the only difference between the variant 4 transcript and that of V3 mentioned above (Figure 6A; Table 3). At the protein level, the amino acid linker between the end of the FNIII domain and the start of the TM domain of RPTPζ-V4 is only 383 amino acids long (Figure 6B; Table 3). The full *Ptprz1*-V4 transcript, which Fujikawa et al. (2017) named *Ptprz*-B, comprises 5,537 nt and encodes a 1,463-amino acid protein.

***Ptprz1*-V5 [NM_001361349]—Exon 12“a”, *Δ*exon 16, exon 21“a”.** The only difference between the V5 transcript and that of V4 is the omission of exon 16 (Figure 6A; Table 3). Exon 16 encodes 7 amino acids (TLKEFYQ) within the helix-turn-helix “wedge” segment of the D1 phosphatase domain, which may be important in the allosteric modulation of phosphatase activity or in interactions with adjacent phosphatase domains (Bilwes et al., 1996; Figure 6B). The full *Ptprz1*-V5 transcript, which Fujikawa et al. (2017) named *Ptprz1*-BΔ*ex16*, comprises 5,471 nt and encodes a 1,456-amino acid protein.

***Ptprz1*-X3 [XM_006505014]—Exon 12“a”, *Δ*exon 16, exon 21“a”.** The NCBI RefSeq database also predicted the hypothetical transcript variant *Ptprz1*-X3, which is identical to V3 except for the lack of exon 16. Although listed by NCBI as hypothetical, Fujikawa et al. (2017) in their Figure 1B had included this transcript as *Ptprz1*-AΔ*ex16,* which they detected in the mouse brain (Figure 6A; Table 3). The full *Ptprz1*-X3 transcript comprises 8,018 nt and encodes a 2,305-amino acid protein.

Finally, at the time that the present investigation commenced, the NCBI RefSeq nucleotide database cataloged four other hypothetical *Ptprz1* transcript variants that, if they were assembled as mature mRNAs and translated, would yield transmembrane RPTPζ variants with all the essential components: a signal peptide, CALD, FNIII, TM domain, and D1 and D2. We discuss these hypothetical constructs next.

***Ptprz1*-X1 [XM_006505012]—Exon 12“a”, exon 16, exon 21“b”.** The only difference between the X1 transcript and that of V3 is the use of exon 21“b” rather than 21“a” (Figure 6A; Table 3). If *Ptprz1*-X1 were expressed and translated, the inclusion of exons 12“a” and 16 indicates that it would possess both the longer 1,232-amino acid extracellular FNIII-TM linker and the larger “wedge” motif in the intracellular D1 phosphatase domain (Figure 6B; Table 3). The utilization of exon 21“b” would encode (compared to exon 21“a”) six additional residues' N-terminal to the catalytic site of D1. The full *Ptprz1*-X1 transcript would comprise 8,285 nt and encode a 2,318-amino acid protein.

***Ptprz1*-X2 [XM_006505013]—Exon 12“a”, *Δ*exon 16, exon 21“b”.** The only difference between the X2 transcript and that of X1 (immediately above) is the omission of exon 16. (Figure 6A; Table 3). The only difference between the X2 transcript and that of X3 is the use of exon 21“b” rather than 21“a” (Figure 6A; Table 3). The absence of exon 16 from this variant predicts that the D1 phosphatase domain would exhibit the smaller “wedge” motif, and the presence of 21“b” would produce the 6-amino acid insert N-terminal to the catalytic domain (Figure 6A; Table 3). The full *Ptprz1*-X2 transcript would comprise 8,264 nt and encode a 2,311-amino acid protein.

***Ptprz1*-X4 [XM_006505015]*—*Exon 12b, exon 16, exon 21“b”.** The only difference between the X4 transcript and that of V4 is the use of exon 21“b” rather than 21“a” (Figure 6A; Table 3). The inclusion of exon 12“b” would produce the shorter extracellular FNIII-TM linker; exon 16 would encode the larger “wedge” motif in D1; and exon 21“b” would produce the 6-amino acid insert near the catalytic domain (Figure 6B; Table 3). The full *Ptprz1*-X4 transcript would comprise 5,510 nt and encode a 1,469-amino acid protein.

***Ptprz1*-X5 [XM_006505017]—Exon 12“b”, *Δ*exon 16, exon 21“b”.** The only difference between the X5 transcript and that of V5 is the use of exon 21“b” rather than 21“a” (Figure 6A; Table 3). The only difference between the X5 transcript and that of X4 is the absence of exon 16. Thus, the protein would have the shorter extracellular FNIII-TM linker (because of exon 12“b”), smaller “wedge” motif in the intracellular D1 (lack of exon 16), and six extra residues near the catalytic site (exon 21“b”; Figure 6B; Table 3).

*Ptprz1*-X1, *Ptprz1*-X2, *Ptprz1*-X4, and *Ptprz1*-X5 contain an alternatively spliced form of exon 21 that we propose to name exon 21“b”. Exon 21“b” is the full-length exon 21 variant, as it does not use the splice site located 18 nt into the exon. We named it 21b because at the beginning of the present investigation, all RPTPζ variants with verified expression used the shorter form of exon 21, which we propose to name exon 21“a”. That is, exon 21“b” had only been present in hypothetical assemblies. RPTPζ products of exon 21“b” containing transcripts will have an extra LSELFQ motif in the D1 phosphatase domain adjacent to the N-terminal side of the catalytic site (Figure 6B).

##### Differential detection of the nine *Ptprz1* transcript variants in the three mouse HC preparations

To determine which of the above described *Ptprz1* transcripts express in mixed neuron–astrocyte HC cultures, P0–P2 pup HC tissue, or adult HC tissue, we prepared cDNA from these three sources and then used GSPs (Table 2) in a series of PCRs designed to amplify sets of cDNA fragments that (with sequencing verification) are diagnostic for each of the *Ptprz1* variants.

***Ptprz1*-V1 (Exons 1–12).** Figure 6C shows that by using the ζEx1_Fwd′ and ζEx12_Rev′ GSPs, we amplified 5,565-bp cDNA fragments from P0–P2 pups and adults, but not from mixed neuron–astrocyte cultures. Cloning these amplicons into the pCR-Blunt plasmid yielded two colonies from P0–P2 pup and two additional colonies from adult HC samples.

Sequencing of the first cloned P0–P2 pup HC amplicon [PP524764] revealed a *Ptprz1*-V1 transcript with two minor variations compared to the consensus [NM_011219] RefSeq entry: (a) omission of nucleotides ^186^CTCTCT^191^ within the exon-1/5′-UTR and (b) a non-synonymous T→C point mutation at exon 1/nucleotide 319 of the consensus sequence that would result in a L4P amino acid substitution in the RPTPζ-V1 signal peptide.

Sequencing of the second cloned P0–P2 pup HC amplicon [PP524765] reveals that this *Ptprz1*-V1 clone contains three differences compared to the consensus [NM_011219] RefSeq entry: (a) omission of nucleotides ^185^CTCTCT^191^ from the exon-1/5′-UTR; (b) a non-synonymous G→A point mutation in exon 3 that would result in an E91K mutation in the translated protein; and (c) retention of a 1,017-nt intron between exon 9 and exon 10, which would result in translation of a truncated 375-amino acid RPTPζ variant that would contain only the first 66% of FNIII. The final four C-terminal residues after L371 would be -VTIR*.

Sequencing of the first cloned adult HC amplicon [PP524766] revealed that it is identical to consensus [NM_011219] RefSeq entry with the exception of a synonymous T→C point mutation in exon 12 at position T^3720^ of the consensus sequence (thus the codon for residue Threonine-1138 would change from ACT to ACC).

Sequencing of the second cloned adult HC amplicon [PP524767] revealed that compared to [NM_011219], this clone lacks nucleotides ^190^CT^19^ ^1^ from the exon-1/5′-UTR. We found no other changes within the ORFs of either of the adult clones, which we predict would result in the expression of an RPTPζ-V1 protein with an amino acid sequence identical to that of the RefSeq consensus [NP_035349].

**Exon 1–30 variants (*Ptprz1*-V3 to *Ptprz1*-V5 and *Ptprz1*-X1 to *Ptprz1*-X5).** For our first-round PCRs, our GSPs were ζEx1_Fwd and ζEx30_Rev, which should amplify all transcripts except *Ptprz1*-V1 (which contains exon 12). Using these products as templates, we performed second-round nested PCRs using the GSP combinations outlined in Table 2. We then determined which of the remaining possible validated or hypothetical *Ptprz1* variants are present in each of the three HC preparations.

***Ptprz1*-V3 vs. *Ptprz1*-X3 (Exon 12“a”, ±exon 16, exon 21“a”).** PCRs primed with ζEx12a-13_Fwd and ζEx20-21a_Rev (Table 2) amplified ∼800-bp cDNA fragments from mixed HC neuron–astrocyte cultures, P0–P2 pups, and adults (Figure 6D, “V3 or X3” lanes). Cloning and sequencing of the ∼800-bp cDNA fragments determined that 69% (9/13) of the cloned amplicons from the cultures were 820-bp fragments from *Ptprz1*-V3 transcripts (i.e., containing exons 12“a”, 16, and 21“a”; [PP524793]–[PP524801]. The remaining 31% (four colonies) were 799-bp fragments of *Ptprz1*-X3 transcripts (exons 12“a”, Δ16, 21“a”; [PP524840]–[PP524843].

Regarding the pups, 60% (3/5) of the clones contain the 820-bp fragment specific for *Ptprz1*-V3 [PP524802]–[PP524804], and 40% (2/5) contain the 799-bp fragment specific for *Ptprz1*-X3 [PP524844]–[PP524845].

Regarding the adults, 67% (4/6) of the clones contain the 820-bp fragment specific for *Ptprz1*-V3 [PP524805]–[PP524808] and 33% (2/6) contain the 799-bp fragment specific for *Ptprz1*-X3 [PP524846]–[PP524847].

***Ptprz1*-V4 vs. *Ptprz1*-V5 (Exon 12“b”, ±exon 16, exon 21“a”).** PCRs primed with ζEx12b-13_Fwd and ζEx20-21a_Rev (Table 2) amplified ∼800-bp cDNA fragments from mixed HC neuron–astrocyte culture cDNA, P0–P2 pups, and adults (Figure 6D, “V4 or V5” lanes). Cloning and sequencing the ∼800-bp cDNA fragments established that 80% (12/15) of the cloned amplicons from the cultures were 826-bp fragments from *Ptprz1*-V4 transcripts (exons 12“b”, 16, and 21“a”; [PP524809]–[PP524820]). The remaining 20% (three colonies) were 805-bp fragments from *Ptprz1*-V5 transcripts (exons 12“b”, Δ16, and 21“a”; [PP524829]–[PP524831]).

Regarding the pups, 50% (5/10) of the clones contain the 826-bp fragment specific for *Ptprz1*-V4 [PP524821]–[PP524825] and 50% (5/10) contain the 805-bp fragment specific for *Ptprz1*-V5 [PP524832]–[PP524836].

Regarding the adults, 50% (3/6) of the clones contain the 826-bp fragments specific for *Ptprz1*-V4 [PP524826]–[PP524828] and 50% (3/6) contain the 805-bp fragment specific for *Ptprz1*-V5 [PP524837]–[PP524839].

*Ptprz1*-X1 vs. *Ptprz1*-X2 (Exon 12“a”, ±exon 16, exon 21“b”). PCRs primed with ζEx12a-13_Fwd and ζEx20-21b_Rev (Table 2) amplified ∼800-bp cDNA fragments from mixed HC neuron–astrocyte cultures, P0–P2 pups, and adults (Figure 6D, “X1 or X2” lanes). Cloning and sequencing of the ∼800-bp cDNA fragments determined that 50% (6/12) of the cloned amplicons from the cultures were 827-bp fragments from *Ptprz1*-X1 transcripts (exons 12“a”, 16, and 21“b”; [PP524784]–[PP524789]). One of the above six cloned amplicons [PP524784] lacks the thymine-5529 nucleotide of the [XM_006505012] consensus sequence. This deletion would cause a frame shift and premature stop in the RPTPζ ORF, 10 amino acids downstream from W1559 (at the end of the transmembrane domain). The remaining predicted residues would be RKCFQTAHF^I* [where ^ designates the location of the frameshift; I (i.e., isoleucine) is an abnormal residue; and * is the C-terminus]. The truncated protein would comprise only 1,669 amino acids. The other five of six 827-bp *Ptprz1*-X1 transcript fragments ([PP524785]–[PP524789]) are identical to the [XM_006505012] consensus sequence. The remaining 50% of the cloned amplicons (six colonies) were 806-bp fragments from *Ptprz1*-X2 transcripts (exons 12“a”, Δ16, 21“b”; [PP524768]–[PP524773]).

Regarding the pups, 33% (3/9) of the clones contain the 827-bp fragment specific for *Ptprz1*-X1 [PP524790]–[PP524792] and 67% (6/9) contain the 806-bp fragment specific for *Ptprz1*-X2 [PP524774]–[PP524779].

Regarding the adults, 100% (4/4) of the clones contain the 806-bp fragment specific for *Ptprz1*-X2 [PP524780]–[PP524783].

***Ptprz1*-X4 vs. *Ptprz1*-X5 (Exon 12“b”, ±exon 16, exon 21“b”).** Finally, PCRs primed with ζEx12b-13_Fwd and ζEx20-21b_Rev (Table 2) amplified ∼800-bp cDNA fragments from mixed HC neuron–astrocyte cultures, P0–P2 pups, and adults (Figure 6D, “X4 or X5” lanes). Cloning and sequencing of the ∼800-bp cDNA fragments determined that 100% (three colonies) of the cloned amplicons from the cultures were 833-bp fragments from *Ptprz1*-X4 transcripts (exons 12“b”, 16, and 21“b”; [PP524848]–[PP524850]).

Regarding the pups, 9% (2/22) of the clones contain the 833-bp fragment specific for *Ptprz1*-X4 [PP524851]–[PP524852] and 91% (20/22) contain the 812-bp fragment specific for *Ptprz1*-X5 (exons 12“b”, Δ16, and 21“b”; [PP524857]–[PP524876]).

Regarding the adults, 20% (4/20) of the clones contain the 833-bp fragment specific for *Ptprz1*-X4 [PP524853]–[PP524856] and 80% (16/21) contain the 812-bp fragment specific for *Ptprz1*-X5 [PP524877]–[PP524892].

#### RPTPζ protein expression in HC neurons and astrocytes in mixed culture, pup, and adult tissue

##### Mixed neuron–astrocyte cultures

**FIGURE 7:**
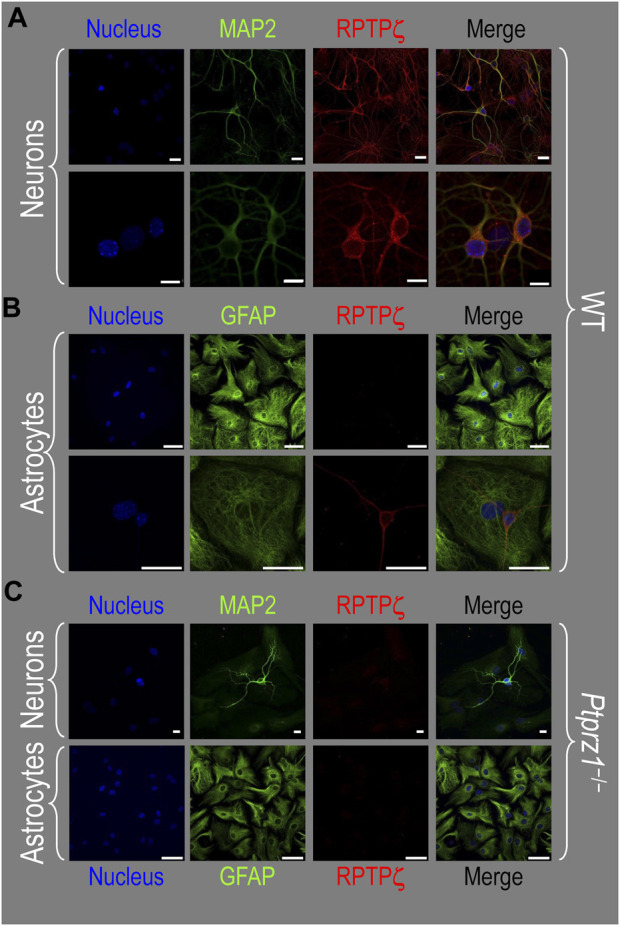
RPTPζ protein expression in HC neurons and astrocytes in mixed culture from WT and *Ptprz1^-/-^* mice. **(A)** Neurons in representative low-magnification (top row) and high-magnification (bottom row) images of mixed neuron–astrocyte HC cultures from WT mice. The columns of panels (from the left) show DAPI staining (blue) of cell nuclei, MAP2 staining (green) to identify neurons, RPTPζ staining (red), and the merge. Note that both the somata and processes of the green MAP2-positive cells (i.e., neurons) stain for RPTPζ (red). **(B)** Astrocytes in representative low-magnification (top row) and high-magnification (bottom row) images of mixed neuron–astrocyte HC cultures from WT mice. The protocol and presentation are the same as in panel A, except that here we used GFAP staining (green) to identify astrocytes. Note that the green GFAP-positive cells (i.e., astrocytes) lack the red RPTPζ staining, which (in this image) we observed only in an adjacent neuronal soma and its processes at high magnification (bottom row). **(C)** Neurons and astrocytes in representative images of mixed neuron–astrocyte HC cultures from *Ptprz1^-/-^* mice. Here, we validated our in-house RPTPζ rabbit polyclonal antibody. The protocol and presentation are the same as in **(A, B)**. Note that none of the cells in these *Ptprz1^-/-^* cultures stained with the red RPTPζ antibody. Scale bars in all panels represent 10 μm.

About 14 days after initiating cultures from WT P0–P2 mouse pups, we identified cells with DAPI and neurons with the MAP2 mouse monoclonal antibody (Figure 7A, green). We observed RPTPζ expression throughout the soma and cellular projections in these MAP2-positive cells (i.e., neurons) by counterstaining using a novel RPTPγ rabbit polyclonal primary antibody (red) that we developed against an extracellular RPTPζ epitope between the FNIII domain and transmembrane domain. In parallel cultures labeled by an anti-GFAP to identify the astrocytes (Figure 7B, green), we observed that RPTPζ (red) co-stained cells shaped like neurons but never did the RPTPζ signal colocalize within the GFAP-positive cells (i.e., astrocytes).

To validate the specificity of our new RPTPζ antibody, we performed immunocytochemical analyses on mixed neuron–astrocyte HC cultures similar to those above but derived from P0–P2 *Ptprz1* ^−/−^ pups. By employing the same staining conditions that we used to visualize RPTPζ expression in WT neurons (Figure 7A) but not RPTPζ astrocytes (Figure 7B), here in the cultures from *Ptprz1* ^−/−^ tissue (Figure 7C), we did not observe significant cross-reaction of our novel RPTPζ antibody with other proteins expressed in these mixed neuron–astrocyte HC cultures.

##### P0–P2 pup HC tissue

**FIGURE 8:**
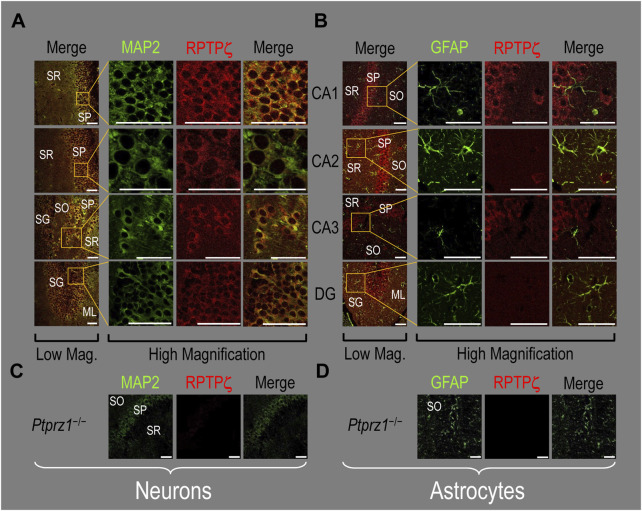
RPTPζ protein expression in P0–P2 pup HC tissue from WT and *Ptprz1^-/-^* mice. **(A)** Neurons in representative examples of low- and high-magnification images of RPTPζ immunostained tissue from WT pups. The columns of panels (from the left) show (a) low-magnification merged images of MAP2 staining (green; individual images not shown) and RPTPζ staining (red; individual images not shown), and then three columns of high-magnification zoomed-in images representing (b) MAP staining (green), (c) RPTPζ staining (red), and (d) the merge. The rows of panels (from the top) show images of the CA1, CA2, CA3, and DG regions. In the low-magnification images, the gold squares delineate ROIs, into which we zoomed-in the three higher-resolution panels to the right. Within the CA regions, we annotate the SO, SP, and SR. Within the DG, we annotate the ML and SG. Note that the neuronal MAP2 marker (green) colocalizes with RPTPζ (red), as indicated in yellow in the merged images, acquired at both low and high magnifications. **(B)** Astrocytes in representative examples of low- and high-magnification images of RPTPζ immunostained tissue. The presentation is the same as in **(A)**, except that here we used GFAP staining (green) to identify astrocytes. Note that the green GFAP-positive cells and processes (i.e., astrocytes) in the CA1–3 regions and DG lack the red RPTPζ staining. The magnified ROIs further highlight that only the neuropil surrounding GFAP-positive cells (i.e., presumably neurons) faintly stains for RPTPζ. **(C)** Neurons in representative examples of low-magnification images of RPTPζ immunostained tissue from *Ptprz1^-/-^* pups. Here, we validate our in-house RPTPζ rabbit polyclonal antibody. The protocol and presentation are the same as in **(A)**. **(D)** Astrocytes in representative examples of low-magnification images of RPTPζ immunostained tissue from *Ptprz1^-/-^* pups. The protocol and presentation are the same as in **(B)**. Scale bars in all panels represent 10 μm.

In the next step, we examined RPTPζ expressed in four regions of P0–P2 pup HC tissue (CA1, CA2, CA3, and DG). In all four HC regions, the MAP2-positive cells (i.e., neurons, green) co-stained for RPTPζ (red), as indicated in yellow in merged images (Figure 8A). In the CA1–CA3 regions, RPTPζ staining is the strongest in the neuronal somata of the SP and also in some somata throughout the neuropil of the SO and SR. Likewise in the DG, we observe RPTPζ staining in the neuronal somata, especially in the SG and, to a lesser extent, ML (Figure 8A).

In parallel sections, we stained astrocytes with the GFAP antibody (Figure 8B, green) and observed no RPTPζ colocalization (red) with GFAP-positive (i.e., astrocytic) somata or projections throughout the CA1, CA2, CA3, or DG. RPTPζ-positive staining is very diffuse in the surrounding neuropil and likely represents adjacent neuronal projections.

When we stained HC sections obtained from P0–P2 *Ptprz1* ^−/−^ pups following the same protocols that we used to stain sections from WT pups, we observed no significant cross-reaction of our novel RPTPζ antibody with other proteins expressed in either neurons (Figure 8C) or astrocytes (Figure 8D).

##### Adult HC tissue

**FIGURE 9:**
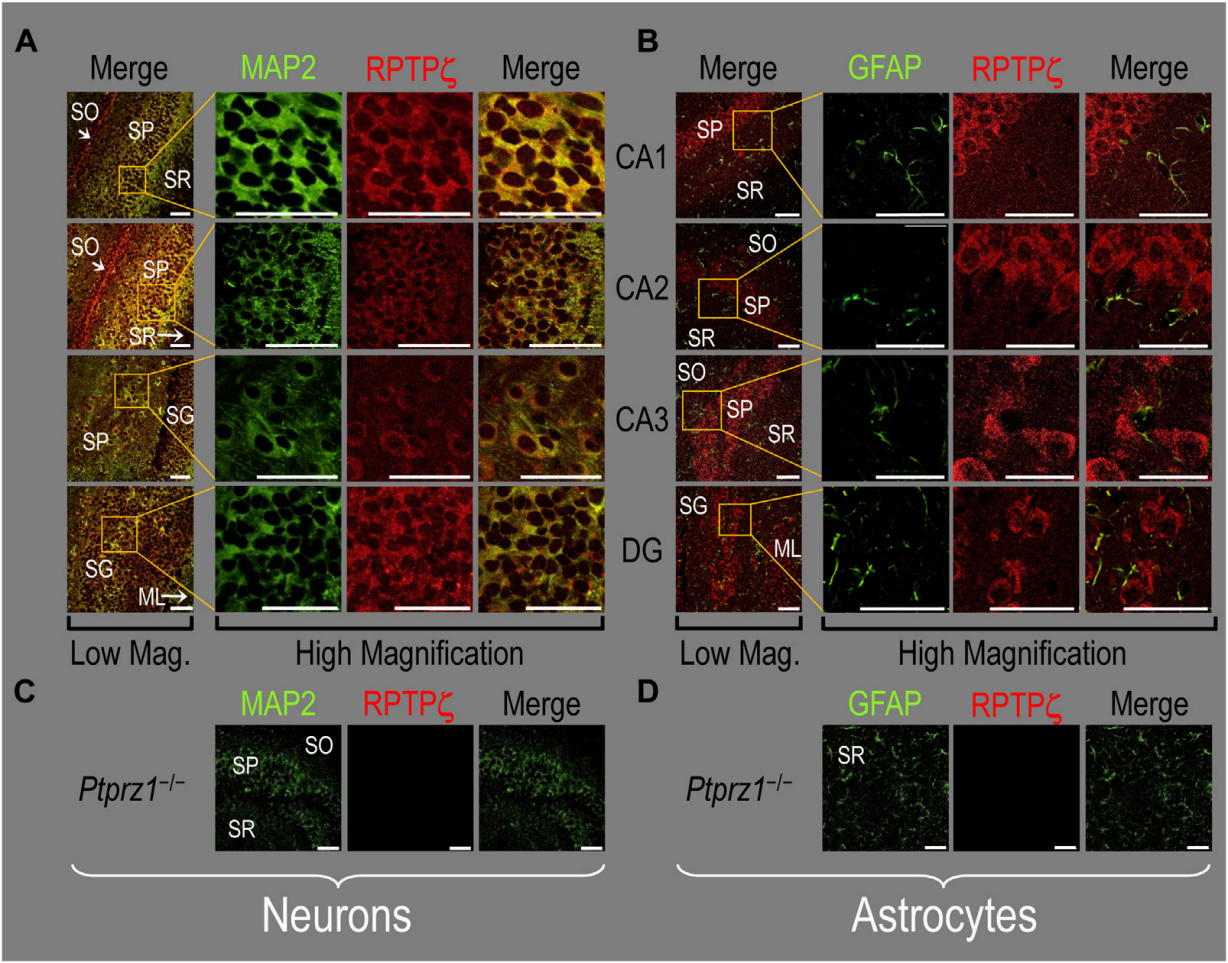
RPTPζ protein expression in adult hippocampal tissue from WT and *Ptprz1^-/-^* mice **(A)** Neurons in representative examples of low- and high-magnification images of RPTPζ immunostained tissue from WT mice. The columns of panels (from the left) show (a) low-magnification merged images of MAP2 staining (green; individual images not shown) and RPTPζ staining (red; individual images not shown), and then three columns of high-magnification zoomed-in images representing (b) MAP staining (green), (c) RPTPζ staining (red), and (d) the merge. The rows of panels (from the top) show images of the CA1, CA2, CA3, and DG regions. In the low-magnification images, the gold squares delineate ROIs, into which we zoomed-in the three higher-resolution panels to the right. Within the CA regions, we annotate the SO, SP, and SR. Within the DG, we annotate the ML and SG. Note that, in the merged images, acquired at both low and high magnifications, the neuronal MAP2 marker (green) colocalizes with RPTPζ (red), as indicated in yellow, particularly in the neuronal soma and the surrounding neuropil. Axonal projections from pyramidal neurons (highlighted by arrows) stain red for RPTPζ and not yellow, most likely because MAP2 distribution is primarily in neuronal soma and dendrites and not axons (Huber and Matus, 1984). **(B)** Astrocytes in representative examples of low- and high-magnification images of RPTPζ immunostained tissue. The protocol and presentation are the same as in **(A)**, except that here we used GFAP staining (green) to identify astrocytes. Note that the green GFAP-positive cells and processes (i.e., astrocytes) in the CA1-3 regions and DG lack the red RPTPγ staining that appears only in neighboring neuronal processes. The magnified ROIs further highlight that only the neuropil surrounding GFAP-positive cells (i.e., presumably neurons) faintly stains for RPTPζ. **(C)** Neurons in representative examples of low-magnification images of RPTPζ immunostained tissue from *Ptprz1^-/-^* mice. Here we validate the rabbit RPTPζ antibody. The protocol and presentation are the same as in **(A)**. **(D)** Astrocytes in representative examples of low-magnification images of RPTPζ immunostained tissue from *Ptprz^-/-^* mice. The protocol and presentation are the same as in **(B)**. Scale bars in all panels represent 10 μm.

In all four regions of adult HC tissue (Figures 9A, B), RPTPζ appears in MAP2-positive cells (i.e., neurons) but not in GFAP-positive cells (i.e., astrocytes), generally following patterns reminiscent of those noted above for tissue from P0–P2 pups.

We detected no significant cross-reaction of the RPTPζ antibody with other proteins expressed in HC sections from adult *Ptprz1* ^−/−^ mice (Figures 9C, D).

### Colocalization of RPTPγ and RPTPζ

The ICC and IHC data presented thus far all indicate that HC neurons but not astrocytes express both RPTPγ and RPTPζ. To validate these observations and determine if RPTPγ and RPTPζ are present in the same neurons and the same cellular compartments within these neurons, we co-stained mixed neuron–astrocyte HC cultures, P0–P2 pup HC sections, and adult HC sections with RPTPγ and RPTPζ antibodies.

#### Mixed neuron–astrocyte HC cultures

**FIGURE 10:**
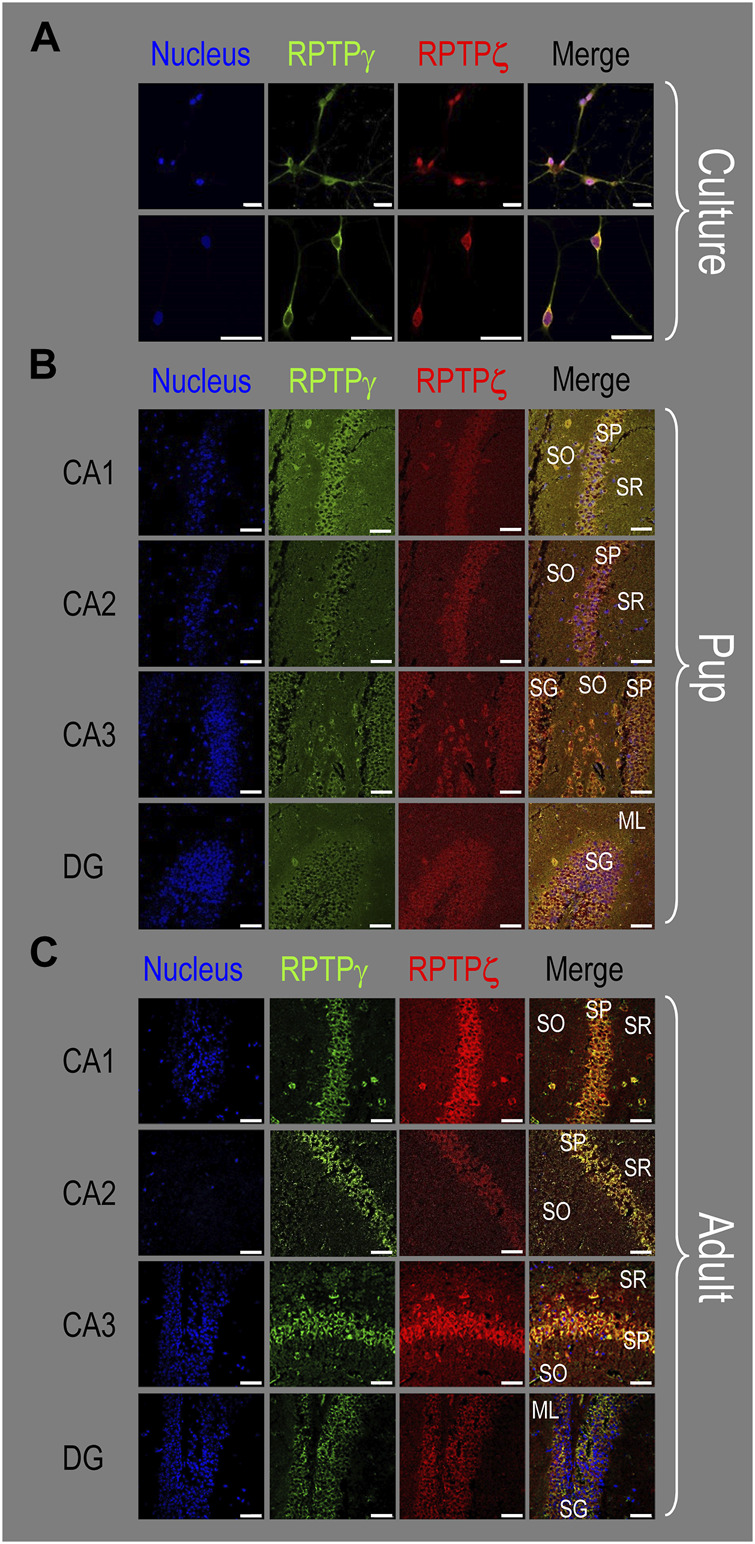
Colocalization of RPTPγ and RPTPζ protein in hippocampal mixed cultures, P0–P2 pup tissue, and adult tissue from WT mice. **(A)** Representative images of mixed neuron–astrocyte HC cultures. The columns of panels (from the left) show images of DAPI staining (blue) of cell nuclei, RPTPγ staining (green), RPTPζ staining (red), and the merge. The upper row is at low magnification, and the lower row is at high magnification. Note the colocalization of the RPTPγ and RPTPζ staining, both at low and high magnifications. **(B)** Representative low-magnification images of P0–P2 pup HC tissue. Within the CA regions, we annotate the SO, SP, and SR. Within the DG, we annotate the ML and SG. The presentation of the columns is the same as in **(A)**. Note the colocalization of RPTPγ and RPTPζ staining, with particularly strong signals in the SP of CA1-3 and in the SG of DG. **(C)** Representative low-magnification images of adult HC tissue. The protocol and presentation are the same as in **(B)**. The colocalization of the RPTPγ and RPTPζ staining is similar to that for the pup tissue in **(B)**. Scale bars in all panels represent 10 μm.

Figure 10A shows that in co-cultures, RPTPγ (green) and RPTPζ (red) are entirely colocalized within the same neurons, as highlighted by yellow in the merged images. Although these particular images may suggest that the RPTPγ antibody may react more strongly with the neuronal processes than the RPTPζ antibody, an examination of the individual staining in Figure 3A (RPTPγ) and Figure 7A (RPTPζ) suggests to us that both antibodies react with neuronal processes.

#### P0–P2 pup HC tissue

Consistent with the individual staining patterns for RPTPγ or RPTPζ in MAP2-positive cells (i.e., neurons) for the pup tissue in Figures 4, 8, we observe in Figure 10B a strong colocalization of the RPTPγ and RPTPζ reactivity at the cellular level in all four HC regions.

#### Adult HC tissue

Again, consistent with the individual staining patterns for RPTPγ or RPTPζ in MAP2-positive cells (i.e., neurons) for the adult tissue in Figures 5, 9, we observe strong colocalization of RPTPγ and RPTPζ at the cellular level (Figure 10C).

## Discussion

In this study, we investigate the expression of *Ptprg* and *Ptprz1* variants in three mouse HC preparations: mixed neuron–astrocyte cultures, P0–P2 pups, and adults. Two of these preparations (HC cultures and adult HC tissue) are widely used by others in the field, and the third represents the source material for HC cultures. For the first time, we validate the expression in mouse of one previously hypothetical *Ptprg* assembly and five *Ptprz1* assemblies that were hypothetical at the initiation of the present study.

We also determine the differential distribution of RPTPγ or RPTPζ protein in the three different mouse HC preparations. Importantly, we show that the vast majority of neurons co-express RPTPγ and RPTPζ in all three HC preparations. However, we find that HC astrocytes lack detectable RPTPγ or RPTPζ.

### RPTPγ variants and expression

#### Confirmation of RPTPγ-V1 and validation of *Ptprg*-X1 as *Ptprg*-V“3”

In all three HC mouse preparations—cultures, pups, and adults—we detect the expression of *Ptprg*-V1 (Figure 1; Table 4), which corresponds to the originally cloned full-length mouse *Ptprg* transcript (Barnea et al., 1993). However, the *Ptprg*-V2 transcript (Table 4) is absent from all three murine HC preparations. *Ptprg*-V2 likely represents an embryonic variant because its transcript validation in the NCBI RefSeq database (Pruitt et al., 2012) is based on mRNA sourced from 11-day-old embryonic spinal cord [AK144283] or 12- to 14-day-old embryonic whole eye [CF724833].

**TABLE 4:** Comparison of mouse validated and hypothetical *Ptprg* and *Ptprz1* transcript assemblies with their human and rat orthologues. The left third of the table lists one mouse (*Mus musculus*) *Ptprg* or *Ptprz1* transcript variant per row, with columns reporting from left to right the variant name, its NCBI, and if available, Ensembl accession numbers (with the common beginning of each group of accession numbers in the column heading), transcript length, and open reading frame (ORF) boundaries. The center and right-most thirds of the table align the validated or hypothetical human (*Homo sapiens*) and rat (*Rattus norvegicus*) orthologues with the corresponding mouse variant. The hypothetical PTPRG-X1, X2, or X3 transcripts have two accession numbers each. The first accession number is predicted from the Homo sapiens chromosome 3, GRCh38.p14 Primary Assembly (NC_000003), and the second is predicted from the CHM13 chromosome 3, alternate assembly T2T-CHM13v2.0 (NC_060927). The hypothetical human PTPRG-X4 and -X5 transcripts are predicted to have different 5′ UTRs, but would encode the same RPTPg-X4 protein (see Table 5). *Ptprg-V3*, *Ptprz1*-V0, and *Ptprz1*-V2 are our proposed designations for newly validated variants.

*Mus Musculus*							*Homo sapiens*							*Rattus norvegicus*						
Gene Name	Variant Name	NCBI Transcript Accession	Ensembl Transcript (ENSMUST)	Transcript Length	ORF Start	ORF Stop	Gene Name	Variant Name	NCBI Transcript Accession	Ensembl Transcript (ENST000)	Transcript Length	ORF Start	ORF Stop	Gene Name	Variant Name	NCBI Transcript Accession	Ensembl Transcript (ENSRNOT)	Transcript Length	ORF Start	ORF Stop
*Ptprg*	V1	NM_008981	00000248537	9190	696	5024	PTPRG	V1 or A	NM_002841	00474889	9357	718	5055	*Ptprg*	V1	NM_134356	00000042010	5130	113	4441
	V2	NM_001347593		6265	94	2097									X6	XM_063273950	00000099333	8736	2580	4583
	X1 or V3	XM_006517956		9225	817	5058		V2	NM_00137547	00295874	9720	718	4968		X3	XM_063273947	00000101353	8445	51	5292
								X1	XM_017006961/XM_054347409		9477	718	5175							
								X2	XM_017006962/XM_054347411		8694	16	4392							
								X3	XM_017006963/XM_054347410		9390	718	5088							
								X4	XM_047448645		12879	4588	8577							
								X5	XM_047448646		9112	821	4810							
															A	AY177703		4478	112	4392
															B	AY177704		4391	112	4305
															C	AY177705		3721	112	3507
															S	AY177706		2581	112	2154
															X1	XM_063273945		8595	15	4442
															X2	XM_063273946		8508	15	4355
															X4	XM_063273948		8059	16	3906
															X5	XM_063273949		8114	152	3961
																	00000088214			
																	00000104654			
*Ptprz1*	V1	NM_011219	00000202579	7062	297	5135	PTPRZ1							*Ptprz1*	V2	NM_001170685		6844	106	4956
	V3	NM_001081306	00000090568	8039	297	7235		V1	NM_002851	00393386	8103	340	7287		V1 or A	NM_013080	00000008719	7871	106	7056
	V4	NM_001311064	00000202102	5492	297	4688		V2	NM_001206838	00651065	5523	340	4707		X2 or B	XM_006236138		5579	388	4779
	V5	NM_001361349		5471	297	4667		V3	NM_001206839	00449182	5502	340	4686		X3	XM_006236139		5558	388	4758
	V6	NM_001403052		8018	297	7214		V4	NM_001369395	00652298	8082	340	7266		X1	XM_006236137		8124	395	7324
	V7	NM_001403053		5510	297	4706														
	V8	NM_001403054		5489	297	4685														
	X1 or V0	XM_006505012		8285	525	7481														
	X2 or V2	XM_006505013		8264	525	7460														
								V5	NM_001369396		8206	485	7390							

Importantly, our amplification and cloning from all three mouse HC preparations of cDNA corresponding to the previously hypothetical [XM_006517956] transcript serves as the first evidence to validate and reclassify the hypothetical *Ptprg*-X1 mRNA assembly in the RefSeq database as *Ptprg*-V“3” (Figure 2; Table 4).

#### Mammalian orthologs of mouse hippocampal RPTPγ variants

**Human.** Early characterization of the human *PTPRG* transcripts identified 6.2 and 9.6 kb mRNAs in normal fetal and adult lungs, kidneys, the digestive tract, pancreas, and spleen (Tsukamoto et al., 1992). The cloning of partial (Kaplan et al., 1990; Krueger and Saito, 1992) and full-length human *PTPRG* cDNA (Barnea et al., 1993) led to the observations that the gene comprises 30 exons on chromosome 3p21–p14 (LaForgia et al., 1991; Kastury et al., 1996) and that *PTPRG* has two major variants. RPTPγ-V1 (or RPTPγ-A, Tables 4, 5) is the longest protein and utilizes all 30 *PTPRG* exons in its ORF. The dominantly expressed shorter form, RPTPγ-V2 (or RPTPγ-B, Tables 4, 5), lacks 29 cytosolic juxtamembrane amino acids (encoded by exon 14) compared to RPTPγ-V1 (Sorio et al., 1995). RefSeq predicts five other hypothetical *PTPRG* assemblies that have no hypothetical or verified mouse orthologs (Tables 4, 5).

**TABLE 5:** Comparison of mouse RPTP**γ** and RPTP**ζ** protein variants with their human and rat orthologues. The left third of the table lists one mouse (*Mus musculus*) RPTPγ or RPTPζ protein variant per row, with columns reporting from left to right the variant name, its NCBI, and, if available, *Ensembl* accession numbers (with the common beginning of each group of accession numbers in the column heading) and protein length. The center and right-most thirds of the table align the same rows of validated or hypothetical human (*Homo sapiens*) and rat (*Rattus norvegicus*) orthologues with the corresponding mouse variant. The hypothetical RPTPγ-X1, X2, or X3 variants have two accession numbers each because, as described in Table 4, the hypothetical transcripts that would encode these variants derive from the primary NC_000003 or alternative NC_060927 human genome assemblies. The human RPTPγ-X4 protein has two accession numbers because its ORF is predicted to reside within either the hypothetical human PTPRG-X4 or -X5 transcripts, as described in Table 4. RPTPγ-V3, RPTPζ-V0, and RPTPζ-V2 are our proposed designations for newly validated variants.

*Mus Musculus*					*Homo Sapiens*					*Rattus norvegicus*				
Protein Name	Variant Name	NCBI Protein Accession	Ensembl Protein (ENSMUSP)	aa	Protein Name	Variant Name	NCBI Protein Accession	Ensembl Protein (ENSP000)	aa	Protein Name	Variant Name	NCBI Protein Accession	Ensembl Protein (ENSRNOP)	aa
RPTPγ	V1	NP_033007	00000159543	1442	RPTPγ	V1 or A	NP_002832	00418112	1445	RPTPγ	V1	NP_599183	00000039045	1442
	V2	NP_001334522		667							X6	XP_063130020	00000095910	667
	X1 or V3	XP_006518019		1413		V2 or B	NP_001362400	00295874	1416		X3	XP_063130017	00000087710	1413
						X1	XP_016862450/XP_054203384		1485					
						X2	XP_016862451		1458					
						X3	XP_016862452/ XP_054203386		1456					
						X4	XP_047304601/XP_047304602		1329					
											A	AAN72429		1426
											B	AAN72430		1397
											C	AAN72431		1168
											S	AAN72432		717
											X1	XP_063130015		1475
											X2	XP_063130016		1446
											X4	XP_063130018		1296
											X5	XP_063130019		1269
													73305	
													94306	237
RPTPζ	V1	NP_035349	144605	1612	RPTPζ					RPTPζ	V2	NP_001164156		1616
	V3	NP_001074775	088056	2312		V1	NP_002842	377047	2315		V1 or A	NP_037212	08719	2316
	V4	NP_001297993	143902	1463		V2	NP_001193767	499073	1455		X2 or B	XP_006236200		1463
RPTPζ	V5	NP_001348278		1456	RPTPζ	V3	NP_001193768	410000	1448	RPTPζ	X3	XP_006236201		1456
	V6	NP_001389981		2305		V4	NP_001356324	499137	2308		X1	XP_006236199		2309
	V7	NP_001389982		1469										
	V8	NP_001389983		1462										
	X1 or V0	XP_006505075		2318										
	X2 or V2	XP_006505076		2311										
						V5	NP_001356325		2301					

**Rat.** RPTPγ-V1 is the only validated rat variant presently in the RefSeq database, and it is the ortholog of both mouse and human RPTP-V1 (Tables 4, 5). Shintani et al. (1997) have cloned cDNAs of four other rat variants (i.e., RPTPγ-A, RPTPγ-B, RPTPγ-C, and RPTPγ-S) that have no correlates, for either mouse or human, among the validated or hypothetical RefSeq or *Ensembl* transcripts (Table 4). RPTPγ-A, RPTPγ-B, and RPTPγ-C lack a 24-nt cassette within exon 16 that encodes the eight amino acids, ^833^HIGELYSN^840^, which in rat RPTPγ-V1, reside four residues before the start of D1. RPTPγ-A and RPTPγ-B also lack another 24-nt cassette within exon 28 that encodes the eight residues, ^1313^CPKWPNPD^1321^, which are located within D2 of rat RPTPγ-V1. This second difference is not present in the 1,176-amino acid RPTPγ-C because it lacks D2 and the C-terminus. The 717-amino acid secreted form, RPTPγ-S (Tables 4, 5), lacks the TM domain and everything afterward and has no ortholog in mice or humans.

The *Ensembl* database predicts three other hypothetical rat *Ptprg* assemblies:

• *Ensembl* [ENSRNOT00000088214]/[ENSRNOP00000073305] that is unlikely to express because it contains only a partial ORF.
• *Ensembl* [ENSRNOT00000104654]/[ENSRNOP00000094306], for which the ORF predicts a secreted 237-amino acid variant due to alternative 3′ exon utilization compared to RPTPγ-S.
• *Ensembl* [ENSRNOT00000099333]/ENSRNOP00000095910] that is also predicted by NCBI RefSeq as *Ptprg*-X6.

*Ptprg*-X6 represents the rat ortholog of mouse RPTPγ-V2 (Tables 4, 5), which has no validated or hypothetical ortholog in humans. Nevertheless, this variant is also predicted in other laboratory mammals, such as

• rabbit (*Ensembl* [ENSOCUT00000047595]/[ENSOCUP00000038485]),
• guinea pig (*Ensembl* [ENSCPOT00000035902]/[ENSCPOP00000023893]), and
• Chinese hamster (*Ensembl* [ENSCGRT00015023958]/[ENSCGRP00015019444]).

Finally, NCBI RefSeq predicts four other hypothetical rat transcripts: *Ptprg*-X1, *Ptprg*-X2, *Ptprg*-X3, *Ptprg*-X4, and *Ptprg*-X5. Our newly defined mouse RPTPγ-V“3” is the ortholog of human RPTPγ-V2 and rat RPTPγ-X3, but no ortholog exists in mouse or human for rat *Ptprg*-X1, *Ptprg*-X2, *Ptprg*-X4, and *Ptprg*-X5 (Tables 4, 5).

#### Other potential RPTPγ variants

During the identification of *Ptprg* as a candidate tumor-suppressor gene, the analysis by Wary et al. (1993) of malignancy-inducing homozygous deletions in murine L-cell sarcoma cell lines (Sanford et al., 1948) showed that the *Ptprg* gene in these cells is missing a 200-kb fragment that includes exons 3–5 and flanking intronic sequence. L-cells faithfully transcribe the mutated gene, which is not a true splice variant, such that the translated mouse RPTPγ mutant lacks amino acid residues 64 through 205. This missing fragment corresponds to a part of the CALD, specifically residues that are homologous to the catalytic site of an active α-CA. We propose that this 142-amino acid deletion renders the resulting RPTPγ mutant incapable of binding CO_2_ or HCO_3_^−^ normally or of transmitting CO_2_/HCO_3_^−^ signals to the intracellular D1 and D2 domains. It appears that a rise in extracellular [CO_2_] or a fall in [HCO_3_^−^] promotes RPTPγ monomerization (Moss et al., 2018), which in renal proximal tubules correlates with increased acid extrusion (Zhou et al., 2016). By analogy with RPTPγ, if the 142-amino acid deletion within RPTPγ in L-cells promotes monomerization, it would raise intracellular pH and promote the malignant phenotype.

Two human cell lines—ACC-LC-171 non–small-cell lung carcinoma and the U-2 OS osteosarcoma—each transcribe the normal 6.2-kb and 9.6-kb human *PTPRG* mRNAs, but each also generates two additional mRNAs (Tsukamoto et al., 1992). These authors detected the expression of extra 4.5-kb and 6.8-kb mRNAs in ACC-LC-171 human non–small-cell lung carcinoma cells and of 7.6-kb and 10.8-kb mRNAs in U-2 OS osteosarcoma cells, detected by using Northern blot analysis using a 2.8-kb *Eco*RI fragment of a partial *PTPRG* cDNA clone (Krueger and Saito, 1992). These results are consistent with the deletion of section(s) of the *PTPRG* gene or of aberrant splicing that yields the additional transcripts in these malignant cells. We did not detect any evidence for the expression of mouse orthologs of these unusual RPTPγ variants in our mouse HC preparations.

#### Expression of RPTPγ in hippocampus

**Neurons.** Our ICC and IHC analyses showing exclusive colocalization of RPTPγ with MAP2-positive staining aligns with previous reports that RPTPγ expression is almost exclusively restricted to neurons both in primary cultures and the healthy CNS (Barnea et al., 1993; Lamprianou et al., 2006; Vezzalini et al., 2007; Lorenzetto et al., 2014). When RPTPγ was cloned, an *in situ* hybridization analysis conducted on the adult rat brain revealed that the highest transcript expression levels are in the hippocampus, particularly the SP (Barnea et al., 1993). We note that our RPTPγ antibody comes from the Sorio laboratory (Mafficini et al., 2007), and our overlapping RPTPγ data agree well with those of the same group (Lorenzetto et al., 2014), specifically that in mouse hippocampus, RPTPγ is strongly expressed in the SP and DG MLs, with lower levels detected in the surrounding neuropil (Lamprianou et al., 2006; Lorenzetto et al., 2014).

**Glial cells.** In the present investigation, we do not observe RPTPγ colocalization with GFAP-positive stained cells (i.e., astrocytes) in mixed neuron–astrocyte HC cultures (Figure 3). This result is in agreement with an earlier report from mixed primary cultures of cortical glial cells (without neurons) based on studies with a β-galactosidase insertion into the *Ptprg* gene that RPTPγ is not expressed in astrocytes but is only detected at low levels in the microglia and oligodendrocytes (Lamprianou et al. (2006). We did not perform counterstaining for microglia or oligodendrocytes in the present study.

In pup (Figure 4) and adult HC tissue sections (Figure 5), we similarly do not observe RPTPγ expression in GFAP-positive cells. This is mostly consistent with a previous report on adult mouse brain tissue sections from several regions (such as the cortex, cerebellum, and hippocampus) in which RPTPγ expression was absent from almost all astrocytes in sections obtained from healthy mice. In the rare cases of RPTPγ positivity in astrocytes, the signal largely correlated with cell size and the degree of GFAP expression: small astrocytes with thin GFAP-positive processes were RPTPγ negative, whereas some larger astrocytes, such as those of HC or perivascular astrocytes, with thick GFAP-positive processes, were RPTPγ positive (Lorenzetto et al., 2014). The minor differences between the present study (i.e., no detected RPTPγ staining in astrocytes) vs the previous one (RPTPγ staining in sparse, large astrocytes) might reflect technical differences. In the present investigation, we stain 5-μm cryosections mounted on SuperFrost-coated slides, whereas Lorenzetto et al. (2014) stained free-floating 30-μm cryosections. The thicker tissue sections may have contributed to the detection of large and relatively rare RPTPγ-positive astrocytes by Lorenzetto et al. (2014). Although both groups used C57BL/6 mice, Lorenzetto et al. (2014) used C57BL/6J, whereas we used C57BL/6_Case_.

We did not study inflammation in the present investigation, but it has been reported that neuroinflammatory stimuli, which include the cytokines TNFα and IL1, can induce RPTPγ transcription in astrocytomas (Schumann et al., 1998). Indeed, nearly all activated HC astrocytes from a 6-month-old 5×FAD Alzheimer’s disease (AD) mouse model are RPTPγ positive (Lorenzetto et al., 2014).

The fraction of microglia that are RPTPγ positive (Mic_PTPRG) is higher in the brains of AD patients vs non-diseased brains (Zou et al., 2024). These Mic_PTPRG interact with both excitatory and inhibitory neurons via CNTN-4 and promote RPTPγ upregulation in the targeted neurons. This upregulation has a potentially neuroprotective role, as the RPTPγ interacts with and activates the vir-like N6-methyladenosine (m^6^A) methyltransferase–associated protein (VIRMA) to block mitophagy-mediated neuronal death in AD or AD models.

### RPTPζ variants and expression

#### Confirmation or validation of *Ptprz* variants

##### Validation of two mouse RPTPζ variants from RefSeq

Comparable to Fujikawa et al. (2017), we detected the expression of *Ptprz1*-V1, *Ptprz1*-V3, *Ptprz1*-V4, and *Ptprz1*-V5 transcripts (Figure 6), in their nomenclature, respectively, *Ptprz*-S, *Ptprz*-A, *Ptprz*-B, *Ptprz*-BΔ*ex16* (Table 3), in almost all of our mouse HC preparations. The notable exception was that *Ptprz1*-V1 is absent from our mixed neuron–astrocyte HC cultures. However, this absence of V1 from the culture aligns with early reports that mature CNS cells secrete RPTPζ-V1/RPTPζ-S/phosphacan (Canoll et al., 1996). Thus, it is possible that our HC cultures are not sufficiently mature to express this variant, even 14–20 days post isolation of the cells from the P0–P2 brain.

Computational analyses of the *Mus musculus* chromosome 6 sequence (NC_000072) can predict hypothetical *Ptprz1* variants. During the course of the present investigation, NCBI performed several updates to the RefSeq database. In the process, NCBI validated the hypothetical assemblies *Ptprz1*-X3 and *Ptprz1*-X4, originally predicted from genomic source sequences [AC133599] and [AC134445]—both RPCI-24 BAC library constructed from male C57BL/6J mouse spleen and/or brain genomic DNA, as expressed variants *Ptprz1*-V6 and *Ptprz1*-V7, respectively (Table 3).

The NCBI probably did not validate the *Ptprz1*-X3 assembly earlier because, although Fujikawa et al. (2017) had previously published their *Ptprz1*-AΔ*ex16* (Table 3) transcript from the mouse brain, the cDNA sequences were not in either the NCBI or *Ensembl* databases. Moreover, the approach of Fujikawa *et al.*, who amplified between exons 12“a” and 18, would not have allowed them to distinguish between *Ptprz1*-X3 and *Ptprz1*-X2. The validation occurred after the submission of transcriptomic evidence for *Ptprz1*-X3 expression from the sequence-read archive (SRA) runs SRR14777531.436575 and SRR14777534.444365, which are a part of the PRJNA667257 and PRJNA547800 BioProjects. PRJNA547800, “Targeted long read sequencing of neuronal cell surface receptor–encoding genes” specifically sources transcript material from the C57BL/6J mouse retina and cerebral cortex. Although NCBI validated *Ptprz1*-X3 as *Ptprz1*-V6 based on, presumably, a single sequence read of one cDNA strand, we now provide independent evidence for *Ptprz1*-V6 mRNA expression in eight clones (four from cultures, two from pups, and two from adults), in which we sequenced both cDNA strands.

NCBI validated *Ptprz1*-X4 as *Ptprz1*-V7 (Table 3) on the basis of its presence in SRR9219380.17411 and SRR9219381.21863, sourced from the C57BL/6J retina and cerebral cortex. Although NCBI validated *Ptprz1*-X4 as *Ptprz1*-V7 based on, presumably, two single-sequence reads of one cDNA strand each, in the present investigation, we now provide independent evidence of *Ptprz1*-V7 mRNA expression in nine clones (three from cultures, two from pups, and four from adults), in which we sequenced both cDNA strands.

#### First report of three previously hypothetical mouse RPTPζ transcript variants

**RPTPζ-X1/V“0”**. Our cloning, from cultures (six clones) and pups (3 clones) but not adults, of cDNA fragments containing *e*xons 12“a”, 16, and 21“b” (i.e., the hypothetical *Ptprz1*-X1) represents the first report, to our knowledge, of a mouse RPTPζ variant that contains exons 12“a”–21“b”. It is possible that RPTPζ-X1 expression is limited to developmental and neonate life stages. We propose designating this variant as *Ptprz1*-V0 because among the Exon 1–30 variants, it (a) contains exons 12“a”, 16, and 21“b”, meaning that its product, RPTPζ-V“0”, would represent the longest possible RPTPζ variant and (b) *Ptprz1*-V1 is already assigned to the lone variant that contains exon 12 (Table 3).

**RPTPζ-X2/V“2”.** We also demonstrate, to our knowledge for the first time, that the *Ptprz1*-X2 hypothetical assembly, identical to *Ptprz1*-X1/V“0” except for the omission of exon 16, is present as mature mRNA in mouse, specifically in HC cultures (six clones), pups (six), and adults (four). We therefore propose redesignating this transcript variant as *Ptprz1*-V2 and the protein as RPTPζ-V2 (Table 3).

**RPTPζ-X5/V8.** Finally, we obtained cDNA clones, not in cultures but from pups (20 clones) and adults (17 clones), comprising exons 12“b”, Δ16, and 21“b”, indicative of the previously hypothetical *Ptprz1*-X5 assembly (Table 3). NCBI recently validated *Ptprz1*-X5 as *Ptprz1*-V8, supported by a single RNA-seq sample (SAMN01164131) from 8-week-old C57BL/6J frontal lobe that on alignment with the mouse chromosome 6 sequence, fully supported all of the predicted introns (Evidence and Conclusion Ontology code ECO: 000348). However, unlike the situations for *Ptprz1*-V6 and *Ptprz1*-V7 (see the previous section “Validation of two mouse RPTPζ variants from RefSeq”), no additional supporting evidence exists at the whole-transcript level. Furthermore, we found no evidence in the published literature reporting detection of mature *Ptprz1*-V8 mRNA. The cloning of the indicative *Ptprz1*-V8 cDNA amplicon from pups and adults in the present study now provides the first strong evidence for its validation. The fact that *Ptprz1*-V8 is not detected in our co-cultured HC neurons and astrocytes suggests that expression of this variant requires factors beyond those present in our culture conditions (Table 3).

#### Mammalian orthologs of mouse HC RPTPζ variants

The present investigation brings nine mouse *Ptprz1* variants with validated expression. By contrast, the validated human *PTPRZ* variants number only five (Table 4).

**Orthologs of murine RPTPζ-V1.** Phosphacan, the secreted RPTPζ variant, was originally isolated and cloned from rat brain (Maurel et al., 1994). This rat extracellular splice variant is now designated *Ptprz1*-V2 in RefSeq and is the rat ortholog of mouse RPTPζ-V1 or RPTPζ-S (Fujikawa et al., 2017). There is no human ortholog of mouse RPTPζ-V1.

**Orthologs of murine RPTPζ-V3**. The human RPTPζ variant 1 (also called PTPζ; Krueger and Saito, 1992) is encoded by *PTPRZ1*-V1. It utilizes a 3,556-nt spliced version of exon 12 (equivalent to the 3,547-nt mouse exon 12“a”) and includes the product of exon 16. Human exon 21 (equivalent to murine exon 21“a”) does not appear to undergo alternative splicing and is present in all validated human variants. The rat *Ptprz1*-V1 transcript is only 1-nt shorter than the human variant and utilizes exons equivalent to both mouse *Ptprz1*-V1 and human *PTPRZ1*-V1 (Table 4). Therefore, both the translated rat RPTPζ-V1, initially called PTPζ-A (Nishiwaki et al., 1998), and the human RPTPζ-V1 are orthologs of mouse RPTPζ-V3 (Table 5). In rats, RPTPζ-V1 (PTPζ-A) expression is stable between ages E13 and P0 and then markedly decreases after birth (Nishiwaki et al., 1998).

**Orthologs of murine RPTPζ-V4.** Human *PTPRZ1*-V2 and rat *Ptprz1*-X2 both include orthologous exons 12“b” and 16, therefore their expression products correspond to mouse RPTPζ-V4 (Tables 4, 5). Although RefSeq lists the rat *Ptprz1*-X2 transcript as hypothetical, the Noda laboratory cloned the cDNA and immunoprecipitated the PTPζ-B rat protein from adult rat brain (Maeda et al., 1994; Nishiwaki et al., 1998).

**Orthologs of murine RPTPζ-V5.** Human *PTPRZ1-V3* and rat *Ptprz1*-X3 include orthologous versions of exon 12“b” but omit exon 16 (Table 4). The translated human RPTPζ-V3 or RPTPζ-X3 proteins therefore correspond to mouse RPTPζ-V5 (Table 5).

**Orthologs of murine RPTPζ-X3/V6.** Human RPTPβ/ζ described by Levy et al. (1993) is RPTPζ-V4, encoded by PTPRZ1-V4 mRNA (Table 4). Because this variant includes the human exon 12 variant corresponding to mouse exon 12“a”, but lacks exon 16, it is the human ortholog of mouse RPTPζ-V6 (formerly RPTPζ-X3; Table 3), which we validated in the present investigation. Rat *Ptprz1*-X4 is also orthologous to mouse RPTPζ-V6 (Tables 4, 5).

**Human PTPRZ1/RPTPζ-V5 (absent in mouse and rat).** Neither human PTPRZ1-V5 nor its product RPTPζ-V5 has a mouse or rat ortholog (Tables 4, 5). Evidence for its expression in humans comes from the SRA SRP049776 in runs SRR1803617.29086 and SRR1803616.62566 (covering the entire transcript assembly) from total brain RNA. *PTPRZ1*-V5 includes the human equivalent of mouse exon 12“b” and omits exon 16. However, the insertion of a novel 31st exon between exons 1 and 2 means that if translation were to initiate at the normal start codon in exon 1, it would terminate prematurely in exon 2, at a stop codon (TAA) that begins 8 nt after the exon 31/exon 2 splice boundary, only yielding a 56-amino acid protein. However, 88 nt into exon 31, there is an alternate start codon in-frame with the normal *PTPRG*Z1 ORF from the exon 31/exon 2 splice boundary onward. Translation from this point will produce a 2,301-amino acid RPTPζ-V5 protein (Table 5). Notably, RPTPζ-V5 does not possess a consensus signal sequence. Consequently, human RPTPζ-V5, if it is expressed, is most likely expressed as an intracellular variant.

As the field continues to validate the expression of additional RPTPγ and RPTPζ variants in various species, it would be helpful, for future new variants and to as great an extent as possible for already validated variants, to standardize the nomenclature across species, to avoid confusion when making comparisons.

#### Expression of RPTPζ in the hippocampus

**Neurons**. RPTPζ is almost exclusively a CNS-expressed protein. Early *in situ* hybridization studies show expression localized in the cerebellar Purkinje cell layer, the DG, and the subependymal layer of the anterior horn of the lateral ventricle (Levy et al., 1993). Subsequent IHC studies in fixed WT adult mouse brain improved the resolution to the cellular level and revealed that RPTPζ protein is detected in cerebellar Purkinje cell dendrites and is also observed as punctate staining along the cortical and HC neuropil (Lorenzetto et al., 2014). In a different study of adult rats that employed both light and electron microscopy, the authors reported that RPTPζ is predominantly present in pyramidal cell dendrites of pyramidal neurons in both the cerebral cortex and HC CA1-3 (Hayashi et al., 2005). At the subcellular level, RPTPζ concentrates as puncta in postsynaptic membranes in some PSD-95–positive spines, both in cortical and HC tissue sections and in cultured cortical neurons (Hayashi et al., 2005). For the HC and hippocampi-derived cultures, in particular, these data are consistent with our findings in the present study that RPTPζ predominantly expresses in MAP2-positive neurons (Figures 7–9).

With respect to the data discussed in the previous paragraph, we observe some differences in localization of the neuronal RPTPζ staining that might either arise due to species differences or reflect the alternative anti-RPTPζ primary antibodies used in ICC and IHC. Regarding species differences, Hayashi et al. (2005) noted that RPTPζ immunoreactivity is greater at apical dendrites than in the somata of rat cortical and HC pyramidal neurons. By contrast, in the present mouse study, we find that somatal RPTPζ expression is strong in culture and distributed evenly through the HC neuron dendrites. Moreover, in tissue sections, HC pyramidal cell somata in the SP have the strongest staining, and staining is not as intense in the SR, where pyramidal cell apical dendrites extend toward the HC fissure (Figures 8, 9). We also observe a prominent stripe of positive (i.e., red) RPTPζ staining within the SO of the CA1 and CA2 regions in adult HC sections in the present study (Figure 9). We suspect that this RPTPζ staining represents axonal projections from the CA1 and CA2 pyramidal neurons. We observe minimal colocalization with MAP2 in these axons because it is known that MAP2 predominantly localizes in neuronal soma and dendrites (the pyramidal cells basal dendrites in the SO) and not in the axons (Huber and Matus, 1984). Regarding antibody differences, our novel rabbit polyclonal RPTPζ antibody, which should recognize all variants, is directed against an extracellular epitope between FNIII and the transmembrane domain, whereas other investigators have used mouse monoclonal antibodies directed against the cytosolic Ct that would not detect RPTPζ-V1 (BD Biosciences Cat# 610180, RRID: AB_397579; Hayashi et al., 2005; Lorenzetto et al., 2014).

**Glial cells.** High RPTPζ expression is observed in radial glial and other glial cell types that play a significant role during development (Canoll et al., 1993; Peles et al., 1995). More recent transcriptomic analyses show that mouse and human astrocytes, respectively, contain the highest *Ptprz1* or *PTPRZ1* mRNA levels in comparison with other classes of glia (Zhang et al., 2014; Zhang et al., 2016). This is in stark contrast with the lack of RPTPζ protein detected in astrocytes in the present study and also observed by others (Lorenzetto et al., 2014). However, it will be necessary to conduct a comprehensive mRNA–protein correlation study to determine whether there is a specific correlation between the abundance of particular *Ptprz1* variant mRNAs and the amount of their translation products in astrocytes under a certain condition. The other reasons that we hypothesize for seeing no RPTPζ protein expression in astrocytes is that our HC preparations are neither damaged nor old. In hypertrophic astrocytes, both RPTPζ-V1 and the full-length transmembrane RPTPζ variants are expressed 30 days post traumatic brain injury (McKeon et al., 1999). Glycosylation diversity on RPTPζ in hypertrophic astrocytes appears to regulate RPTPζ expression levels (Takahashi et al., 2023). Furthermore, as animals age, it appears that most astrocytes assume a more reactive phenotype, and RPTPζ transcript levels steadily increase, particularly in the hippocampus (Clarke et al., 2018).

### Co-expression of RPTPγ and RPTPζ in HC neurons but their absence from astrocytes

Some authors report that the expression pattern for RPTPγ is distinct from that of RPTPζ. For example, in one mouse IHC study, RPTPγ was present throughout the neuronal somata and dendritic processes of the cerebral cortex and hippocampus, whereas RPTPζ appeared as punctate staining along the neuropil (Lorenzetto et al., 2014).

In the present HC study, we visualize the expression of both RPTPγ and RPTPζ proteins in virtually every MAP2-positive cell (i.e., neurons), both in the somata and projections, but in virtually no GFAP-positive cells (i.e., astrocytes). We base these conclusions on data from mixed neuron–astrocyte HC cultures (Figures 3, 7) and on HC tissue from pups (Figures 4, 8) and adults (Figure 5, 9). Note that in all preparations, the RPTPγ- and RPTPζ-positive neurons are adjacent to the RPTPγ- and RPTPζ-negative astrocytes, making it unlikely that the negativity of the astrocytes reflects a global degradation of the respective antigens. It is also worth noting that although the primary antibodies in the present investigation are raised against extracellular RPTPγ or RPTPζ epitopes (see Methods section), the abundant intracellular RPTPγ and RPTPζ expression in HC neurons in permeabilized tissue sections suggests the existence of substantial intracellular pools of both proteins. These pools likely play a role in their transportation to or removal from the cell membrane.

Our results are broadly consistent with previous work concluding that RPTPγ and RPTPζ are both largely absent from astrocytes of healthy young adults. However, in contrast to Lorenzetto et al. (2014), who reported that RPTPγ and RPTPζ express in distinct and partially overlapping HC neuron populations, we observe that RPTPγ and RPTPζ expression patterns are virtually superimposable in our cultures, pups, and adults (Figure 10).

## Conclusion

The primary objective of this study was to investigate the splice variants and expression patterns of RPTPγ and RPTPζ proteins in mouse hippocampus, recognizing their potential involvement in the sensing of extracellular [CO_2_] and [HCO_3_^−^] and regulation of acid–base transport in the CNS.

We validate the number of mouse RPTPγ variants at three and RPTPζ variants at nine. We provide the first cDNA evidence for transcription of four of these variants (*Ptprg*-V3, *Ptprz1*-X1/V“0”, *Ptprz1*-X2/V“2”, and *Ptprz1*-X5/V8) and, in most cases (except *Ptprz1*-V1, *Ptprz1*-X1/V“0”, and *Ptprz1*-X5/V8) detect them in all three HC preparations. Other studies have investigated RPTPγ and RPTPζ expression in mouse, human, and rat brain, but as far as we are aware, the present study is the first to emphasize mouse P0–P2 pup HC tissue. The findings of our study align broadly with the previous literature regarding the almost exclusive expression and localization of RPTPγ in neurons. Nevertheless, the current findings differ from some previous studies, which suggest that RPTPγ and RPTPζ express in distinct neuronal types or that RPTPζ is primarily expressed in glial cells. Our work may necessitate a reevaluation of some physiological data reported for cells/tissue isolated from the brains of *Ptprz1* ^−/−^ mice. Additionally, our work raises the question of why at least HC neurons express both RPTPγ and RPTPζ. Work on kidneys (Zhou et al., 2016) implicates RPTPγ in sensing [CO_2_]_o_ and [HCO_3_^−^]_o_ and the control of acid secretion. Preliminary data on mixed neuron–astrocyte HC cultures (Taki et al., 2023) similarly implicates RPTPζ for impacting intracellular pH homeostasis. Aside from differences in the binding partners of RPTPγ and RPTPζ, it is possible that the two molecules have different sensitivities to CO_2_ vs. HCO_3_^−^, or transduce the signals to different downstream targets.

## Data Availability

The data that support the findings of this study are available from the corresponding author upon reasonable request. cDNA sequences were submitted to GenBank (www.ncbi.nlm.nih.gov) and the accession numbers for every clone are provided in the article.

## Footnotes

1 According to the NCBI, a “validated” sequence is one for which it has been confirmed that “the genomic sequence corresponding to an annotated mRNA feature matches the mRNA sequence record, and that coding region features really can be translated into the corresponding protein sequence” (Pruitt et al., 2012).

2 Throughout the article, we use double quotation marks to enclose proposed exon-21 designations (i.e., those not yet formalized by the NCBI or other bodies). Thus, we write proposed 21a as 21“a”. See Results section for rationale.
